# Antigen spreading mediates heterogeneous solid tumor eradication by DNA demethylating agent–programmed CAR T cells

**DOI:** 10.1126/sciadv.adz4088

**Published:** 2026-05-08

**Authors:** Yelei Guo, Chuan Tong, Jianshu Wei, Zhiqiang Wu, Yuting Lu, Fuxin Han, Yipeng Zhang, Chunmeng Wang, Jinhong Shi, Fengxia Shi, Yao Wang, Weidong Han

**Affiliations:** ^1^ Department of Bio-therapeutic, The First Medical Center, Chinese PLA General Hospital, Beijing 100853, China.; ^2^ Changping Laboratory, Beijing 102206, China.

## Abstract

Antigen heterogeneity substantially limits the efficacy of chimeric antigen receptor–modified T (CAR T) cell therapy against solid tumors. Our study highlights the potent antitumor activity of low-dose decitabine-primed CAR T (dCAR T) cells in solid tumor models, a benefit previously confirmed in hematologic malignancies. Notably, dCAR T cell infusion in immunocompetent mice led to substantial elimination of mixed tumor masses containing both antigen-positive and antigen-negative cells, without the need for prior lymphodepletion. Our analysis showed notable proinflammatory remodeling of the tumor immunosuppressive microenvironment. Crucially, antigen-activated dCAR T cells sustained high levels of interferon-γ production, which induced immunogenic cell death in tumor cells and activated conventional dendritic cells. This, in turn, stimulated endogenous CD8^+^ T cells, enhancing their antigen-spreading capacity and aiding in the clearance of abscopal antigen-negative tumors. These findings reveal the robust antigen-spreading capability of dCAR T cells, underscoring their clinical potential in addressing solid tumors with inherent antigen heterogeneity.

## Teaser

DNA demethylating agent–primed CAR T cells induce antigen spreading, eradicating heterogeneous solid tumors.

## INTRODUCTION

Chimeric antigen receptor–modified T (CAR T) cells have seen rapid advancements in recent years, demonstrating remarkable clinical efficacy in treating B cell hematological malignancies. This success has generated substantial interest in extending CAR T cell therapy to solid tumors (*1*, *2*). Despite numerous studies confirming the safety and feasibility of using CAR T cells for solid tumors, the clinical outcomes remain suboptimal (*3*–*5*). Antigen heterogeneity, characterized by the incomplete expression of the targeted antigen across tumor cells, represents a major challenge to the therapeutic efficacy of CAR T cells, particularly in solid tumor treatment (*6*, *7*). This limitation has been demonstrated in a clinical trial evaluating epidermal growth factor receptor (EGFR)–targeting CAR T cells for pancreatic cancer, where the emergence of EGFR-negative tumor variants was observed (*8*). Similarly, antigen heterogeneity has been documented in patients with relapsed glioblastoma treated with anti-EGFRvIII CAR T cells (*9*). Therefore, there is an urgent need for an effective strategy to address antigen heterogeneity, thereby enhancing the antitumor efficacy of CAR T cells.

Emerging evidences indicate that antigen spreading (AS) plays a crucial role in enhancing antitumor efficacy, with current data underscoring its therapeutic significance (*10*, *11*). AS refers to a phenomenon characterized by the activation of endogenous T cell immunity directed toward nontargeted tumor-associated antigens (TAAs). The priming of antigen-presenting cells (APCs) has been established as an essential prerequisite for AS in T cells, a fundamental process required to induce endogenous T cell immunity (*12*). Following tumor cell lysis under initial therapy, APCs, particularly dendritic cells (DCs), uptake nontargeted TAAs, subsequently cross-presenting these antigens to prime polyclonal T cell responses against residual malignancies. To date, there remains limited evidence suggesting that CAR T therapy itself induces the generation of therapeutically potential AS. Only a few preclinical studies have reported weak antitumor-specific T cell responses associated with this phenomenon (*13*). The modification of CAR T cells to coexpress additional immunostimulatory molecules, such as interleukin-7 (IL-7)/CCL19 (*14*), CD40L (*15*), IL-12 (*16*), and even bacterial virulence factor (*17*), has enabled the observation of enhanced antitumor effects against antigen-positive tumor cells, as well as the documented generation of specific antitumor endogenous T cells observed in immunocompetent murine models. However, further investigation is required to evaluate the efficacy of these modified CAR T cells against heterogeneous relapsed or abscopal tumors. Some studies have reported that CAR T cells combined with stimulator of interferon genes (STING) agonists or synchronous vaccines in the treatment of solid tumors successfully activated AS and induced endogenous antitumor responses in immunocompetent mice, and some abscopal effects were observed (*7*, *18*). Although the use of multiple agents in combination increases the complexity and uncertainty of clinical translation, these animal-based results suggest the potential value of inducing AS to improve the efficacy of CAR T cells in the treatment of heterogeneous solid tumors.

We recently demonstrated that the application of low-dose decitabine [5-aza-2′-deoxycytidine (DAC)], a potent DNA methyltransferase inhibitor, substantially enhanced the antitumor efficacy of CAR T cells against hematological malignancies both in vitro and in immunodeficient xenograft tumor models (*19*). Notably, this DNA programming effect is specifically triggered by the interaction between the chimeric antigen receptor and its target antigen. Moreover, other studies have demonstrated that pharmacological inhibition of DNA methyltransferase or genetic ablation of de novo DNA methyltransferase 3 alpha (*DNMT3A*) can effectively potentiate T cell antitumor efficacy while ameliorating their exhaustion (*20*, *21*). According to this information, we further extended the use of low-dose DAC-programmed CAR T (dCAR T) cells treating solid tumors.

In several syngeneic tumor models using immunocompetent mice, AS was observed following the administration of dCAR T cell therapy. This phenomenon was characterized by a marked increase and activation of endogenous tumor-specific CD8^+^ T cells in the mice, including at the tumor site, spleen, and peripheral blood. These T cells demonstrated the ability to respond to negative antigens, thereby facilitating the control of mixed tumor masses composed of both target antigen-positive and antigen-negative tumor cells, and effectively limiting the growth of abscopal antigen-negative tumor lesions, even without prior lymphodepletion conditioning. We found that dCAR T cells specifically targeted and eradicated antigen-positive tumor cells while simultaneously releasing elevated levels of interferon-γ (IFN-γ; exceeding conventional CAR T outputs), which induced robust immunogenic cell death (ICD) in antigen-negative counterparts. This cascade activated DCs to prime tumor-specific endogenous CD8^+^ T cells, ultimately leading to the eradication of antigen-heterogeneous malignant populations. Unlike engineering approaches or multiagent combination therapies, this study provides a one-stop CAR T cell platform capable of inducing potent antitumor immunity in endogenous CD8^+^ T cells, effectively eradicating antigenically heterogeneous solid tumors.

## RESULTS

### dCAR T cells exhibit enhanced antitumor efficacy and induce a change in the composition of endogenous immune cells within antigen-positive solid tumors

In this study, we first evaluated the antitumor efficacy of dCAR T cells against solid tumor in immunocompetent mice. We generated murine anti-CD19 CAR T cells by transducing murine T cells with a retrovirus, and produced murine dCAR T cells by incorporating a 10 nM DAC during CAR T cell culture, as described in our previous study (fig. S1, A and B) (*19*). Consistent with our previous observations in human dCAR T cells, murine dCAR T cells exhibited no significant difference in terms of cell proliferation and viability, CAR expression, or cell composition compared to conventional CAR T in the “resting state” (fig. S1, C to F). However, there was a significant enrichment of central memory T cells (Tcm, CD44^+^CD62L^+^) in the dCAR T cells compared to conventional CAR T cells (fig. S1F). In the cytotoxicity experiments, cocultured with the melanoma cell line B16F10, which constitutively expresses human CD19 (CD19^+^B16), dCAR T cells showed markedly superior ability to lyse tumor cells (fig. S2, A to C). This enhanced ability in dCAR T cells exhibited increased levels of CD107a expression, cytokine production, Tcm cell population, and Ki67 proliferation index, when compared to conventional CAR T cells (fig. S2, D to J).

**Fig. 1.**
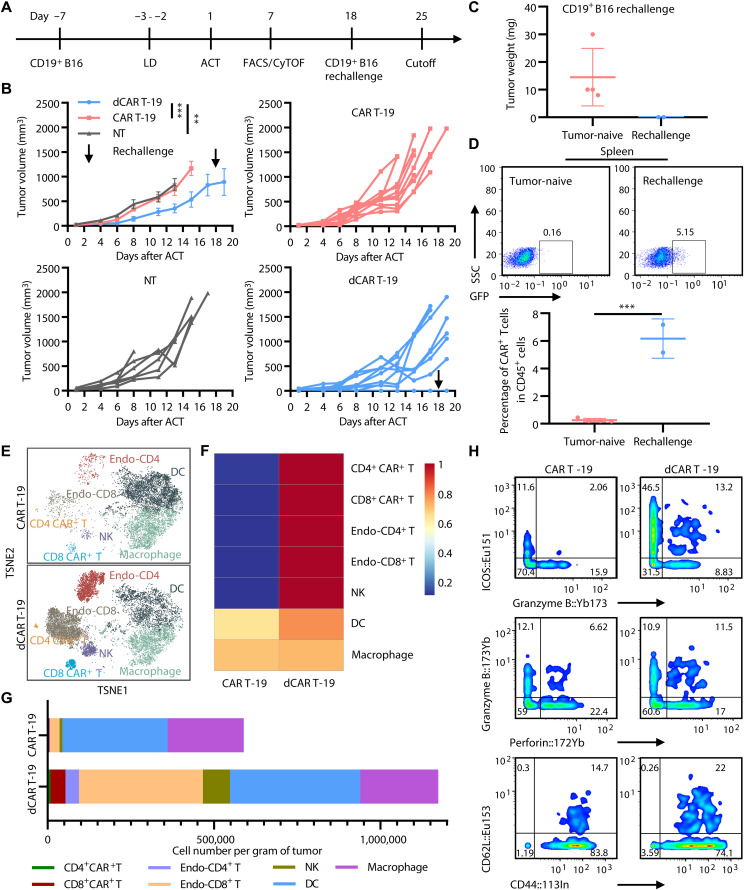
dCAR T cell therapy reshapes the endogenous immune cell landscape. (**A**) Schematic of in vivo experimental design. LD, lymphodepletion; ACT, adoptive cell therapy. (**B**) Line charts depicting general and individual tumor growth in mice bearing CD19^+^B16 tumors after treatment with untransduced T (NT), CAR T-19, and dCAR T-19 cells (NT group, *n* = 6; other groups, *n* = 10). (**C**) Histogram showing tumor weight in mice after rechallenged with CD19^+^B16 cells for 7 days (tumor-naive group, *n* = 4; rechallenge group, *n* = 2). (**D**) Representative flow plots (top) and histogram (bottom) showing the percentage of CAR^+^ T (GFP^+^) cells in CD45-positive splenocytes after CD19^+^B16 tumor rechallenge (tumor-naive group, *n* = 4; rechallenge group, *n* = 2). (**E**) t-distributed stochastic neighbor embedding (TSNE) views indicating distinguishing tumor-infiltrating immune cell subpopulations in mice bearing CD19^+^B16 tumors after ACT for 7 days using mass cytometry analysis. Endo indicates endogenous. (**F**) Heatmap showing the number of tumor-infiltrating immune cells by mass cytometry analysis. The data have undergone standardized normalization processing. (**G**) Histogram from mass cytometry analysis depicting the number of tumor-infiltrating immune cells. (**H**) Contour plots from mass cytometry analysis showing pairwise expression of the indicated markers (top: ICOS and granzyme B; middle: granzyme B and perforin; bottom: CD62L and CD44) on tumor-infiltrating endogenous T cells. Plots gated on CD45.2^+^CD3^+^ cells. Data represent a pool of *n* = 5 samples per group [(E) to (H)]. Statistical significance was determined by two-way ANOVA (B), two-tailed unpaired Student’s *t* test (D). **P < 0.01; ***P < 0.001.

Subsequently, we conducted an in vivo antitumor efficacy evaluation of dCAR T cells. Following tumor establishment via subcutaneous implantation of 3 × 10^5^ CD19^+^B16 cells in immunocompetent mice (Fig. 1A), we administered lymphodepletion by intraperitoneal pro-conditioning of 200 μg of cyclophosphamide and 100 μg of fludarabine (*16*, *22*, *23*). Thereafter, the mice received adoptive cell therapy (ACT) by intravenous injection of 1 × 10^7^ cells. Treatment with dCAR T cells substantially suppressed tumor growth, resulting in complete tumor regression in a subset of mice. In contrast, conventional CAR T cells showed limited efficacy, and tumors in the untransduced T cell (NT) group progressed rapidly (Fig. 1B). Subsequently, we subcutaneously implanted CD19^+^B16 tumor cells into the contralateral flank of the two surviving mice that had achieved complete response in the prior experiment. We found no tumor growth in the rechallenged mice and identified CAR^+^ T cells in their spleens (Fig. 1, C and D). These findings collectively suggest that dCAR T cells have a relatively superior persistent memory to achieve tumor surveillance in vivo.

Immunosuppression of the tumor microenvironment (TME) has been considered as a critical factor contributing to the limited efficacy of CAR T therapy in solid tumors (*24*). We evaluated the status of endogenous immune cells in the TME after CAR T treatment using an animal model of congenic (CD45.1^+^) dCAR T cells infused into C57BL/6 (CD45.2^+^) recipient mice (Fig. 1A). After 7 days of cell infusion, we observed a significant reduction in tumor growth in the dCAR T group (fig. S3A). Using flow and mass cytometry on posttreatment tumor-infiltrating immune cell mixtures, the dCAR T cell treatment group showed a significant increase in tumor-infiltrating CD45^+^ leukocytes (fig. S3B), accompanied by an increase in the number of CAR^+^ T cells, endogenous T cells, natural killer (NK) cells, and DCs compared to the conventional CAR T group (Fig. 1, E to G, and fig. S3, C and D), especially endogenous T cells, which increased 13-fold compared to the conventional CAR T group (Fig. 1G). The number of tumor-infiltrating endogenous T cells in the dCAR T group far exceeded the number of CAR^+^ T in the tumor tissue (7.6-fold) (Fig. 1G). The endogenous T cells concurrently exhibited notable elevated levels of activation (Icos^+^Gran B^+^), effector function (Gran B^+^perforin^+^), and memory phenotypes (CD44^+^CD62L^+^) in the dCAR T group (Fig. 1H), suggesting that the prominent controlling effect of dCAR T on solid tumor may be jointly achieved by CAR T targeting antigen-positive tumor cells combined with the activation of endogenous antitumor efficacy.

### Heterogeneous tumor clearance by dCAR T–triggered endogenous T cells

**Fig. 2.**
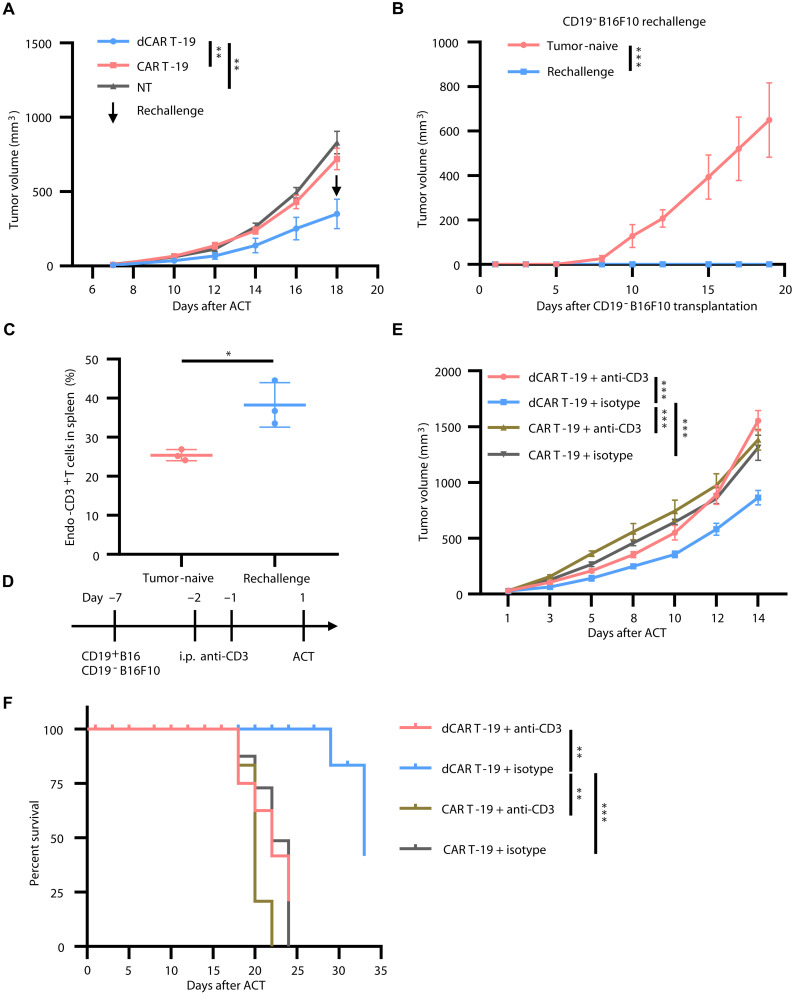
dCAR T cells enable endogenous T cells to eradicate heterogeneous tumor. (**A**) Line chart depicting tumor growth of mice bearing CD19^+^B16 and CD19^−^B16F10 mixed tumors upon ACT (NT group, *n* = 6; other groups, *n* = 10). (**B**) Line chart showing tumor growth in mice after rechallenged with CD19^−^B16F10 cells (tumor-naive group, *n* = 3; rechallenge group, *n* = 3). (**C**) Histogram depicting the comparison of endogenous CD3^+^ T cells in spleen after CD19^−^B16F10 rechallenged (tumor-naive group, *n* = 3; rechallenge group, *n* = 3). (**D**) Schematic of in vivo experimental design for (E) and (F). Mice were intraperitoneally injected with depleting antibodies for CD3^+^ T cells after implantation of a mixture of antigen-negative and -positive tumor cells followed by CAR T cell infusion. (**E**) Tumor growth upon ACT in the presence or absence of depleting anti-CD3 antibodies (*n* = 5). (**F**) Overall survival of mice following ACT (*n* = 5). Statistical significance was determined by two-way ANOVA [(A), (B), and (E)], log rank (Mantel-Cox) test (F), or two-tailed paired Student’s *t* test (C). *P < 0.05; **P < 0.01; ***P < 0.001.

Given that the activation of endogenous T cells is a critical characteristic of AS (*7*), and in light of the aforementioned findings, we further used a mixed tumor model consisting of CD19^+^B16 and B16F10 (CD19^−^B16F10) at a 1:1 ratio to investigate whether dCAR T treatment could activate endogenous T cells and induce an antitumor response against target antigen-negative tumors. First, we determined that CD19^+^B16 and CD19^−^B16F10 cells had comparable growth rates in C57BL/6 mice (fig. S4A). To more comprehensively assess the endogenous immune response following dCAR T cell treatment, we removed the lymphodepletion preconditioning regimen. Unexpectedly, even in the absence of preconditioning, anti-CD19 dCAR T cells were capable of inducing significant regression of CD19-positive and -negative mixed tumors. Thirty percent of mice bearing heterogeneous tumor mixtures achieved complete remission following treatment (Fig. 2A and fig. S4B). In contrast, the conventional CAR T group exhibited minimal control over mixed tumor growth, comparable to the NT group (Fig. 2A and fig. S4B). Next, we performed a CD19^−^B16F10 rechallenge experiment on mice that had achieved complete remission after dCAR T treatment. We observed no new tumor growth for 20 days after tumor cell inoculation, and there was a marked increase in endogenous T cells in the spleens of the rechallenged mice (Fig. 2, B and C). To determine whether exogenous dCAR T or endogenous T cells exerted a clearance effect on CD19-negative tumor cells, we treated the mice with CD3^+^ T cell–depleting antibodies following tumor cell implantation but before CAR T cell infusion (Fig. 2D). When endogenous T cells were blocked, dCAR T cells, in a manner analogous to conventional CAR T cells, showed only a modest degree of inhibition of mixed tumor growth. dCAR T cells were only able to achieve control of antigenically heterogeneous tumors if endogenous T cells were retained (Fig. 2, E and F).

Having identified that endogenous T cells may represent the predominant cell population responsible for eliminating target antigen-negative tumor cells following dCAR T treatment, we further evaluated their functionality and characterization using single-cell transcriptome sequencing. We established a mixed tumor model with a 1:1 ratio of CD19^+^B16 and CD19^−^B16F10 cells and then used the resulting tumor masses to conduct single-cell RNA sequencing (scRNA-seq) 7 days after cell therapy (fig. S5A). Using established cell type–specific gene signatures (*25*), we initially annotated clusters exhibiting similar expression patterns (fig. S5, B and C). Subsequent unsupervised clustering of the transcriptome data revealed five endogenous CD8 and four endogenous CD4 clusters (fig. S5D), and we further categorized these clusters into CD8^+^ (effector or exhaustion), CD4^+^ [effector, exhaustion, or regulatory T cell (T_reg_ cell)], and a few undefined clusters (fig. S5E). The CD8-C2-*Gzmd* and CD4-C1-*Gzma* clusters, both indicative of effector T cells, predominantly comprised cells from tumors treated with dCAR T cells, whereas the CD8-C4-*Tox* (CD8^+^ exhausted T cells), CD4-C4-*Pdcd1* (CD4^+^ exhausted T cells), and CD4-C3-*Foxp3* (CD4^+^ T_reg_ cells) clusters were primarily populated with cells from tumors treated with CAR T cells (fig. S5F). Subsequent Gene Ontology (GO) and Kyoto Encyclopedia of Genes and Genomes (KEGG) analyses revealed that differentially expressed genes within the CD8-C2 and CD4-C1 effector clusters in mice treated with dCAR T cells were substantially enriched in pathways related to immune and inflammatory response, T cell activation and proliferation, regulation of T cell migration, chemokine signaling pathway, and metabolic relative pathways (fig. S5, G to J). These results suggest that dCAR T–treated tumor-infiltrating endogenous T cells exhibit notable activation and proliferation of effector transcriptome features compared to tumor-infiltrating endogenous T cells after conventional CAR T treatment.

### dCAR T cell induces AS of endogenous CD8^+^ T cells

**Fig. 3.**
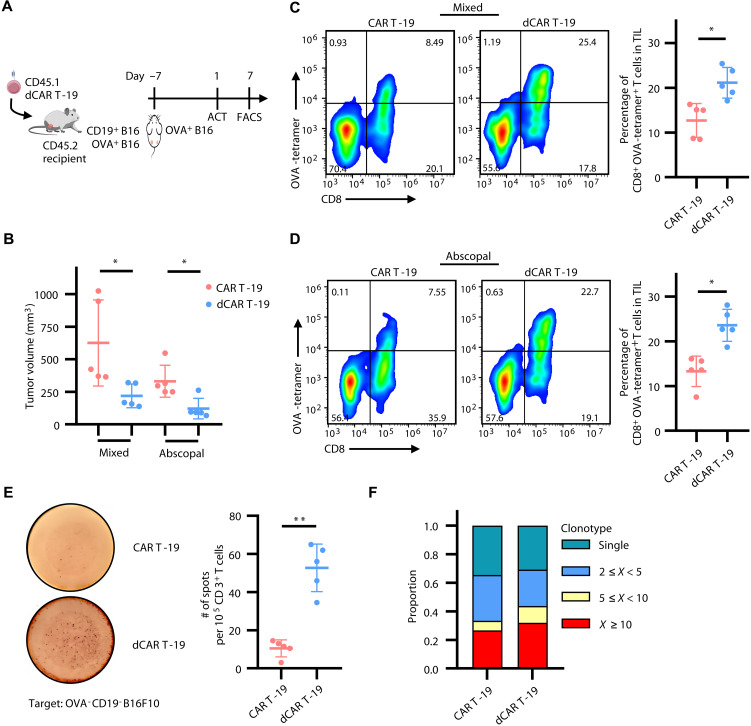
dCAR T cells induce antigen spreading in endogenous CD8^+^ T cells. (**A**) Schematic of in vivo experimental design. Created with biogdp.com. C57BL/6 (CD45.2) mice were administered dCAR T (CD45.1) cell infusion after inoculated with CD19^+^B16 and OVA^+^B16 mixed tumors in the left flank and OVA^+^B16 abscopal tumors in the right flank. (**B**) Histogram showing tumor volumes for mixed and abscopal tumors on day 7 after ACT (*n* = 5). (**C** and **D**) Representative flow plots (left) and histogram (right) showing the frequency of tumor-infiltrating OVA-tetramer^+^ CD8^+^ T cells in mixed (C) and in abscopal tumor masses (D) on day 7 after ACT (*n* = 5). Flow plots gated on CD45.2^+^CD3^+^ T cells. (**E**) IFN-γ ELISPOT (*n* = 5). Endogenous T cells sorted from spleen to coculture with OVA and CD19 double-negative B16F10 tumors. (**F**) Frequencies of TCR clonotypes in endogenous CD8^+^ T cells originating from CAR T-19– and dCAR T-19–treated CD19^+^B16 and OVA^+^B16 mixed tumors on day 7 after ACT. Statistical significance was determined by two-tailed paired Student’s *t* test (B to E). *P < 0.05; **P < 0.01.

Emerging evidence suggests that AS could be a major factor to the therapeutic benefits observed in heterogeneous tumors during cancer immunotherapy (*6*, *17*). Herein, to assay the extent of AS of endogenous T cells after dCAR T cell therapy, we used B16F10 murine melanoma cells expressing the surrogate antigen ovalbumin (OVA^+^B16). We inoculated C57BL/6 mice with a mixture of CD19^+^B16 and OVA^+^B16 in the left flank and OVA^+^B16 alone in the right flank, defining the abscopal tumor lesion (Fig. 3A). The dCAR T cells not only showed remarkable control of mixed tumors but also exhibited outstanding superior inhibition of abscopal CD19-negative and OVA-positive tumors (Fig. 3B). In mice treated with dCAR T cells, there was a marked increase in the percentage of host OVA-specific (SIINFEKL-directed) CD8^+^ T cells within bilaterally engrafted tumors (Fig. 3, C and D). Then, we isolated splenocytes on day 7 and cultured with irradiated OVA^−^CD19^−^ B16F10 cells in an IFN-γ enzyme-linked immunospot (ELISPOT) assay to evaluate the host T cell response against antigens not targeted by CAR T cells. We detected more than 52 T cell immunospots (range from 34.5 to 65) in the dCAR T group after cocultured with CD19 and OVA double-negative tumor cells, far exceeding the 10 (3 to 14.5) immunospots observed in the conventional CAR T group (Fig. 3E). Last, we assessed the change in the T cell receptor (TCR) clonal repertoire of host CD8^+^ T cells from the mixed tumors using scRNA-seq and TCR sequencing, and we observed that the proportion of more than five cell clones was significantly higher in the dCAR T group compared with the CAR T group (Fig. 3F and fig. S6, A to C), indicating an improved TCR oligoclonal amplification of host tumor-reactive CD8^+^ T cells in the dCAR T group related to the CAR T group. Furthermore, we assessed AS of host CD8^+^ T cells directly against TAAs and found a significant increase in host MuLV p15E-specific (KSPWFTTL-directed) CD8^+^ T cells in mice bearing CD19^+^B16 tumors following the treatment with dCAR T cells (fig. S7). This suggests that dCAR T cells can also promote AS in endogenous T cells, enabling them to recognize a broader range of TAAs.

To confirm that AS occurred in the endogenous T cells after dCAR T treatment, we again used the MC38 colorectal cancer cell line for validation. Similarly, we inoculated C57BL/6 mice with a mixture of CD19^+^MC38 and OVA^+^MC38 in the left flank and OVA^+^MC38 alone in the right flank (fig. S8A). Following ACT, we observed remarkable control of both mixed tumors and abscopal tumors (fig. S8, B and C). In addition, we performed an IFN-γ ELISPOT assay with irradiated CD19 and OVA double-negative MC38 cells, revealing significantly more T cell immunospots in the dCAR T group compared with the conventional CAR T group (fig. S8D). We also observed a notable increase in the percentage of host OVA-specific (SIINFEKL-directed) CD8^+^ T cells within bilaterally engrafted tumors in the dCAR T group (fig. S8, E and F). To further explore the potential universal effects of DNA demethylation on ACT, we inoculated mice with a mixture of OVA^+^B16 and OVA^−^B16F10 in the left flank and OVA^−^B16F10 in the right flank, followed by treatment with low-dose DAC-treated CD8^+^ OT-1 T cells (fig. S9A). After ACT, enhanced tumor control was achieved in both mixed and abscopal tumor lesions in the DAC-treated CD8^+^ OT-1 T cell group (fig. S9, B and C). Furthermore, we noted endogenous T cell priming against non-OVA antigens in the DAC-treated group (fig. S9D).

Collectively, these findings indicate that the potential AS in host tumor-reactive CD8^+^ T cells could be induced by dCAR T cell treatment, thereby eradicating abscopal antigen-negative tumor lesions.

### The requirement of activated DCs for AS of endogenous T cells

**Fig. 4.**
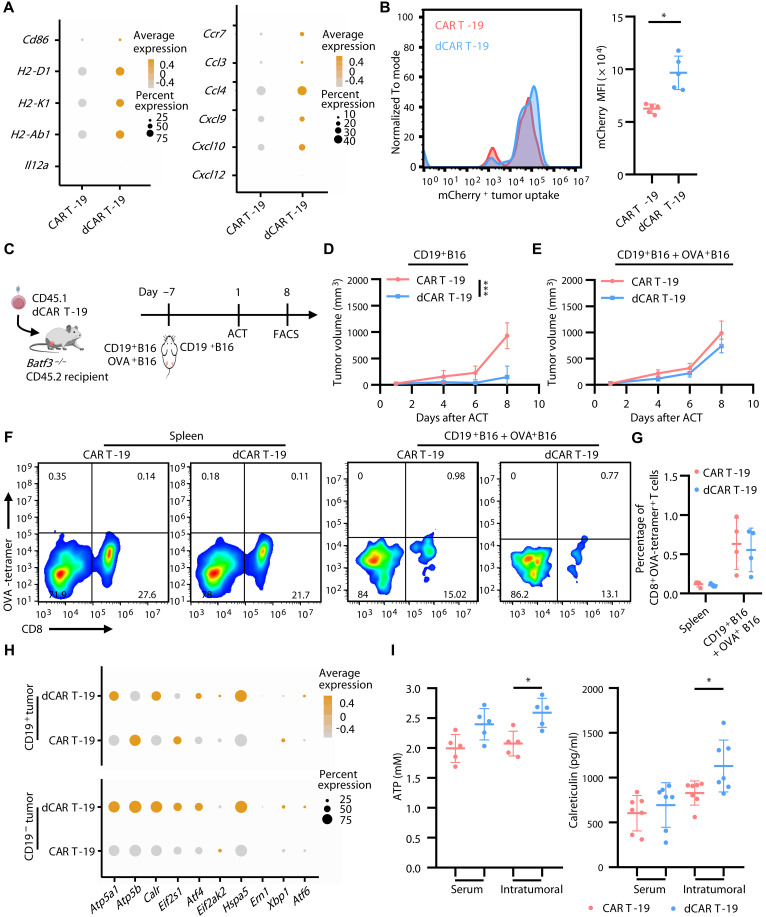
Activated DCs underlie antigen spreading induced by dCAR T therapy. (**A**) Dot plots illustrating differential expression of signature genes in tumor-infiltrating DCs in mice bearing CD19^+^B16 and CD19^−^B16F10 mixed tumors analyzed by scRNA-seq (*n* = 2). (**B**) Representative flow plot (left) and histogram (right) depicting tumor antigen (mCherry) uptake by intratumoral DCs in mice bearing mCherry^+^CD19^+^B16 tumors following the treatment with CAR T-19 and dCAR T-19 cells (*n* = 5). (**C**) Schematic of the in vivo experimental design for (D) to (G). *Batf3*^−/−^ C57BL/6 (CD45.2) mice were administered dCAR T (CD45.1) cell infusion after inoculated with CD19^+^B16 and OVA^+^B16 mixed tumors in the left flank and CD19^+^B16 alone in the right flank. Created with biogdp.com. (**D** and **E**) Line charts showing CD19^+^B16 (D) and mixed CD19^+^B16 and OVA^+^B16 (E) tumor growth in mice after ACT (*n* = 5). (**F** and **G**) Representative flow plots (F) and histogram (G) of endogenous OVA-tetramer^+^ CD8^+^ T cells in spleen and in CD19^+^B16 and OVA^+^B16 mixed tumors from the CAR T-19– and dCAR T-19–treated group on day 8 after ACT (*n* = 4). Flow plots gated on CD45.2^+^CD3^+^ T cells. (**H**) Dot plots illustrating differential expression of signature genes in antigen-positive and -negative tumor cells in mice bearing CD19^+^B16 and CD19^−^B16F10 mixed tumors analyzed by scRNA-seq (*n* = 2). (**I**) Histogram illustrating the expression levels of ATP (*n* = 5) and calreticulin (*n* = 7) in peripheral blood and in CD19^+^B16 and CD19^−^B16F10 mixed tumors on 7 days after ACT in C57BL/6 mice bearing CD19^+^B16 and CD19^−^B16F10 mixed tumors in the left flank and CD19^−^B16F10 cells in the right flank. Statistical significance was determined by two-way ANOVA [(D) and (E)] or two-tailed paired Student’s *t* test [(B), (G), and (I)]. *P < 0.05; ***P < 0.001.

It is well established that AS requires the activation of DCs to take up TAAs and thereby induce endogenous T cell responses (*12*). Therefore, to subsequently establish that dCAR T cells have the ability to induce AS, initial investigations prioritized assessing the potential contribution of DCs to the AS mechanism. Previous flow and mass cytometry analyses of posttreatment tumors had observed a significant increase in the number of tumor-infiltrating DCs in the dCAR T group (Fig. 1, F and G, and fig. S3D). scRNA-seq data revealed an up-regulated gene signature associated with the activation in tumor-infiltrating DCs, including costimulatory molecules such as *MHC I*, *MHC II*, and *Cd86*, in the dCAR T group (Fig. 4A). In addition, we found chemokines such as *Ccr7*, *Ccl3*, *Ccl4*, *Cxcl9*, and *Cxcl10*, which recruit and activate T cells, to be up-regulated (Fig. 4A). Furthermore, we observed an increase in the frequency of DCs responsible for completing antigen presentation, including conventional DC1 (cDC1) and migration DC (migDC), in mice treated with dCAR T cells (fig. S10). Using a CD19^+^B16 tumor cell line expressing mCherry as a traceable antigen, we found that dCAR T therapy could increase tumor antigen uptake by DCs compared to the CAR T group (Fig. 4B), suggesting an increasing antigen-presenting capacity of DCs. Together, these data indicate an increased activation and number of intratumor DCs following dCAR T cell therapy.

Next, we used *Batf3*^−/−^ mice deficient in cross-presenting DCs to determine the acquisition of DCs to drive endogenous T cell priming (Fig. 4C). In these mice, the dCAR T group exhibited superior tumor control in CD19^+^B16 tumor lesions (Fig. 4D) but showed no significant difference in tumor control compared to the CAR T group for the mixed CD19^+^B16 and OVA^+^B16 tumor lesions (Fig. 4E). In addition, OVA-specific CD8^+^ T cells were undetectable in the spleen and in OVA^+^B16 and CD19^+^B16 mixed tumors (Fig. 4, F and G). These results suggest that DC activation induced by dCAR T therapy is an important prerequisite for the establishment of AS.

We then explored the key factors underlying DC activation. scRNA-seq analysis first revealed that the expression levels of danger-associated molecular patterns (DAMPs) such as adenosine triphosphate (ATP; *Atp5a1* and *Atp5b*) and calreticulin (*Calr*) were increased in both antigen-positive and -negative tumor cells in dCAR T therapy group (Fig. 4H). DC recruitment, activation, and antigen uptake could be enhanced by DAMPs released from dying tumor cells that are processed by ICD (*26*, *27*). This was accompanied by significantly increased levels of ATP and calreticulin in serum and tumor homogenates in the dCAR T therapy group (Fig. 4I). In addition, we observed an up-regulation in the expression of genes related to the endoplasmic reticulum stress response such as *Atf4* and *Hspa5* in both antigen-positive and -negative tumor cells in the dCAR T group compared to the conventional CAR T group (Fig. 4H). Together, these data suggest that ICD occurs in tumor cells after dCAR T cell therapy.

To further analyze the ICD induced by dCAR T cells in tumor cells, we cocultured antigen-positive tumor cells with dCAR T cells and evaluated the extent of membrane permeabilization, which facilitates the release of intracellular DAMPs. Flow cytometric analysis using annexin V and 7-aminoactinomycin D staining confirmed that dCAR T cells induced significantly greater membrane permeabilization in antigen-positive tumor cells (fig. S11A). In addition, we detected significant amounts of ATP and calreticulin in the coculture supernatants of the dCAR T cell group (fig. S11B). Tumor cells treated with dCAR T cells showed increased major histocompatibility complex class I (MHC-I) expression (fig. S11, C and D), suggesting that these cells present tumor autoepitopes to endogenous tumor-specific T cells. We also observed increased membrane permeabilization in antigen-negative tumor cells, indicative of bystander killing, which we had physically separated from the coculture of dCAR T and antigen-positive tumor cells using a Transwell (fig. S11, E and F). Together, these data suggest that dCAR T cells induce more extensive ICD in tumor cells, thereby strengthening the prerequisite for DC activation.

### The prominent AS effect is caused by the ICD of target antigen-negative tumor cells due to the sustained high level of IFN-γ production after dCAR T activation

**Fig. 5.**
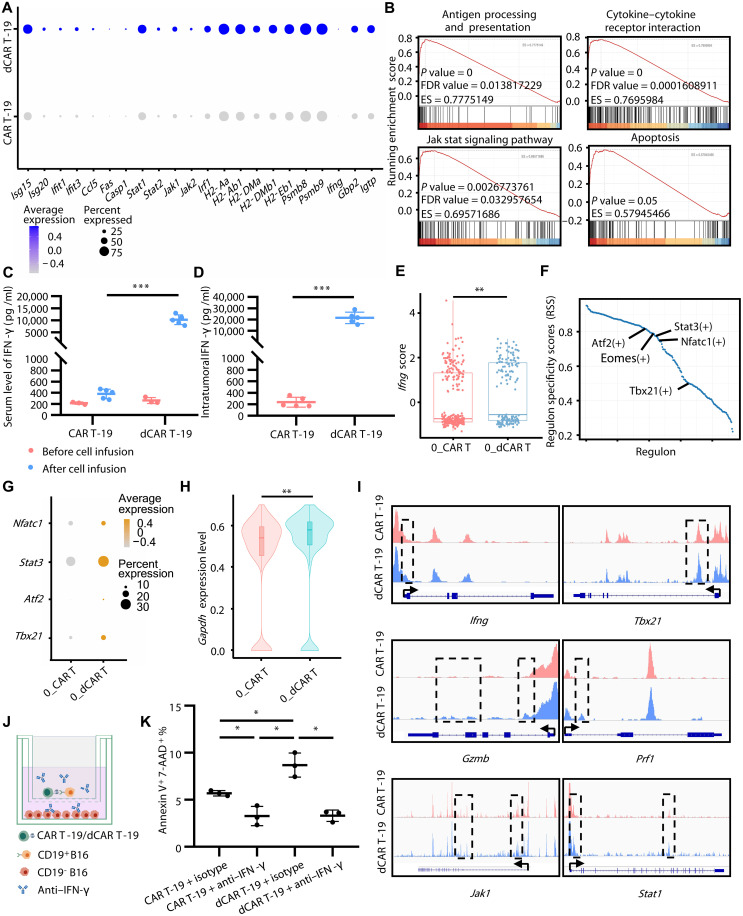
dCAR T cells trigger the immunogenic cell death of antigen-negative tumor cells via IFN-γ signaling. (**A**) IFN-γ–responsive gene expression in antigen-negative tumor cells in mice bearing CD19^+^B16 and CD19^−^B16F10 mixed tumors analyzed by scRNA-seq (*n* = 2). (**B**) Representative GSEA enrichment plots demonstrating the up-regulation of antigen processing and presentation, cytokine-cytokine receptor interaction, JAK-STAT signaling pathway and apoptosis pathways in antigen-negative tumor cells in the dCAR T group versus that in the CAR T group (*n* = 2). (**C** and **D**) The expression levels of IFN-γ in peripheral blood (before group, *n* = 3; after group, *n* = 5) (C) and in mixed tumor masses (*n* = 5) (D) 7 days after ACT in C57BL/6 mice simultaneously bearing CD19^+^B16 and CD19^−^B16F10 mixed tumors and CD19^−^B16F10 abscopal tumors. (**E**) The *Ifng* expression score in 0_CAR^+^ T cluster in mice bearing CD19^+^B16 and CD19^−^B16F10 mixed tumors (0_CAR T group, *n* = 264; 0_dCAR T group, *n* = 151). (**F**) The regulons regulated *Ifng* expression in 0_CAR^+^ T cluster in the dCAR T group versus that in the CAR T group (*n* = 2). (**G**) Differential expression of *Tbx21*, *Atf2*, *Nfatc1*, and *Stat3* in the 0_CAR^+^ T cluster (*n* = 2). (**H**) Gene expression level for *Gapdh* in the 0_CAR^+^ T cluster (0_CAR T group, *n* = 264; 0_dCAR T group, *n* = 151). (**I**) Representative ATAC-seq tracks from in vitro cultured CAR T and dCAR T cells without antigen activation at *Ifng*, *Tbx21*, *Gzmb*, *Prf1*, *Jak1*, and *Stat1* regions (*n* = 2). (**J**) Schematic of in vitro experimental design for (K). Image created with biogdp.com. (**K**) Flow analysis for membrane permeabilization of antigen-negative tumor cells in the presence or absence of anti–IFN-γ antibody (*n* = 3). Statistical significance was determined by two-tailed unpaired Student’s *t* test [(C), (E), and (H)], or two-tailed paired Student’s *t* test [(D) and (K)]. *P < 0.05; **P < 0.01; ***P < 0.001.

We have shown that following dCAR T treatment, target antigen-negative tumor cells undergo ICD, which promotes DC activation. This process induced AS and activated endogenous tumor-specific T cells, leading to the elimination of target antigen-negative tumor cells. However, we also observed that although dCAR T cells has a remarkably enhanced efficacy in killing target antigen-positive tumor cells (Fig. 1), it does not directly induce ICD in heterogeneous tumor cells (Fig. 2, D to F; CD3 antibody blockade); thus, we further investigated the mechanism underlying the initiation of ICD in antigen-negative tumor after dCAR T cell infusion. CD19-negative tumor cells in the dCAR T group exhibited a substantial up-regulation of IFN-γ–responsive genes, including *Ccl5*, *Isg15*, *Stat1*, and *Jak* (Fig. 5A). Furthermore, Gene Set Enrichment Analysis (GSEA) revealed a significant enrichment of antigen processing and presentation, cytokine-cytokine receptor interaction, JAK-STAT signaling pathway, and apoptosis pathways in antigen-negative tumor cells of the dCAR T group compared to that in the CAR T group (Fig. 5B). These results suggest that IFN-γ–responsive gene expression in CD19-negative tumor cells is enhanced following dCAR T treatment, accompanied by the activation of downstream responsive genes and associated signaling pathways.

We analyzed the expression levels of IFN-γ in mice 7 days after ACT, and we found that both serum and intratumoral IFN-γ levels were significantly increased in dCAR T–treated mice compared to those in the conventional CAR T group (Fig. 5, C and D). Moreover, previous in vitro experiments demonstrated that dCAR T cells produced higher levels of IFN-γ upon stimulation with CD19^+^B16 (fig. S2, E and F). Single-cell sequencing of tumor tissue from day 7 after ACT treatment revealed that tumor-infiltrating CAR^+^ T cells in the dCAR T group had significantly elevated levels of *Ifng* expression related to the conventional CAR T group (Fig. 5E and fig. S12), together with up-regulation of key regulators of IFN-γ expression, including *Tbx21*, *Atf2*, *Nfat*, *Stat3*, and *Gapdh* (Fig. 5, F to H) (*28*, *29*). Then, to further determine the DNA-demethylated program-mediated *Ifng* and its response gene expression, we conducted assay for transposase-accessible chromatin with high-throughput sequencing (ATAC-seq) analysis to examine epigenetic regulatory changes in dCAR T cells before infusion. Our findings indicated that ex vivo DAC treatment of CAR T cells resulted in increased chromatin accessibility at the *Ifng* loci and its associated regulator (*Tbx21*), effector (*Gzmb* and *Prf1*), and response (*Stat1*, *Jak1*, *Jak2*, *Irf1*, *Casp1*, *Icam1*, and *Gbp2*) genes before cell infusion (Fig. 5I and fig. S13).

IFN-γ signaling has been reported to play an essential role in the process of cell death (*30*–*32*). Mechanically, IFN-γ can directly induce tumor cell apoptosis or promote nonapoptotic cell death via autophagy, or indirectly sensitize tumor cells to immune responses or chemotherapy-induced apoptosis. On the basis of the abovementioned findings, we propose that high intertumoral IFN-γ levels induce ICD in tumor cells. Transcriptomic profiling and flow cytometry confirmed abundant IFN-γ receptor expression across tested tumor models (B16 and MC38) (fig. S14, A to D). Subsequently, direct in vitro stimulation of tumor cells with recombinant IFN-γ at a concentration of 20 ng/ml, previously detected intratumorally, resulted in significant tumor cell death after 48 hours (fig. S14, E and F). Then, we physically separated antigen-negative tumor cells cocultured with dCAR T cells and antigen-positive tumor cells using a Transwell insert in the presence of IFN-γ–blocking antibodies. In this setting, we observed a decrease in cell death in antigen-negative tumor cells when exposed to the IFN-γ neutralizing antibody (Fig. 5, J and K, and fig. S14G). These data suggest that high-level IFN-γ released by target antigen-activated dCAR T cells may bypass antigen dependence to induce ICD in antigen-negative tumor cells.

**Fig. 6.**
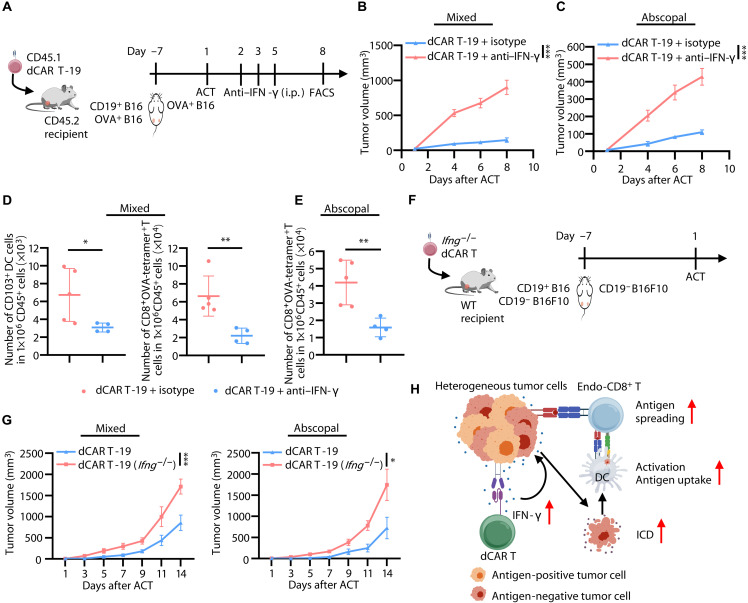
Enhanced IFN-γ production driven by dCAR T cells triggers AS. (**A**) Schematic of in vivo experimental design for (B) to (E). Image created with biogdp.com. C57BL/6 (CD45.2) mice were administered dCAR T (CD45.1) cell infusion after inoculated with CD19^+^B16 and OVA^+^B16 mixed tumors in the left flank and OVA^+^B16 abscopal tumors in the right flank, and subsequently received anti–IFN-γ antibodies. (**B** and **C**) Line charts showing mixed (B) and abscopal (C) tumor growth in mice after ACT (*n* = 5). (**D**) Enumeration of the number of tumor-infiltrating CD103^+^ DCs and endogenous OVA-tetramer^+^ CD8^+^ T cells in mixed tumors (isotype group, *n* = 5; anti–IFN-γ group, *n* = 4).(**E**) Histogram showing the number of tumor-infiltrating endogenous OVA-tetramer^+^ CD8^+^ T cells in abscopal tumors (isotype group, *n* = 5; anti–IFN-γ group, *n* = 4). (**F**) Schematic of in vivo experimental design for (G). Wild-type (WT) C57BL/6 mice were administered *Ifng*^−/−^ dCAR T cell infusion after inoculated with CD19^+^B16 and CD19^−^B16F10 mixed tumors in the left flank and CD19^−^B16F10 abscopal tumors in the right flank. Image created with biogdp.com. (**G**) Line charts depicting mixed (left) and abscopal (right) tumor growth in mice after ACT (*n* = 7). (**H**) Graphical summary depicting that AS is induced by IFN-γ–mediated ICD, which is released from antigen-activated dCAR T cells. Image created with biogdp.com. Statistical significance was determined by two-way ANOVA [(B), (C), and (G)] and two-tailed unpaired Student’s *t* test [(D) and (E)]. *P < 0.05; **P < 0.01; ***P < 0.001.

Furthermore, to determine whether IFN-γ serves as a key initiator of AS, we treated mixed tumor-bearing mice with dCAR T cells in the presence of neutralizing antibody against IFN-γ (Fig. 6A). We found that the administration of IFN-γ–blocking antibodies significantly decreased the serum IFN-γ level (fig. S15A). Although it did not affect CAR^+^ T cell infiltration into the tumors (fig. S15B), this blockade abolished tumor control in CD19^+^B16 and OVA^+^B16 mixed tumor lesions (Fig. 6B). In addition, we observed that the presence of IFN-γ–blocking antibodies impaired tumor control in abscopal OVA^+^B16 tumor lesions (Fig. 6C). We found a notable reduction of CD103^+^ DCs and OVA-specific CD8^+^ T cells infiltrating into treated tumors in the presence of IFN-γ–blocking antibodies in mixed tumor lesions (Fig. 6D). Moreover, blockade of IFN-γ resulted in a pronounced decline of OVA-specific CD8^+^ T cells in abscopal tumor lesions (Fig. 6E). These results suggest that the activation of the IFN-γ signaling pathway is an essential prerequisite for the occurrence of ICD to induce AS.

Last, we determined the source cells of elevated IFN-γ levels within tumor regions after dCAR T therapy. Here, we used T cells derived from *Ifng*^−/−^ mice to generate dCAR T cells, which had lost the capacity for IFN-γ secretion. After the mice bearing CD19^+^B16 and CD19^−^B16 mixed tumors and abscopal CD19^−^B16 tumors received cell infusion, we found that the *Ifng*^−/−^ dCAR T group failed to control both mixed and abscopal tumor lesions, in contrast to the effective control achieved with dCAR T treatment (Fig. 6, F and G, and fig. S15, C and D). In conclusion, we have definitively identified dCAR T cells as the source of intratumoral IFN-γ, which subsequently induced ICD in antigen-negative tumor cells. This cytokine-mediated bystander effect elicits AS, initiating a potent endogenous antitumor immune response and culminating in the eradication of heterogeneous tumors.

## DISCUSSION

Addressing tumor antigen heterogeneity in CAR T cell therapy for solid tumors is critical to advancing the development of effective cancer immunotherapy. Building on our previous work, which demonstrated the durable antitumor activity of dCAR T cells in hematologic malignancies (*19*), we sought to address the additional challenges posed by the greater antigenic complexity and immune evasion capacity characteristic of solid tumors (*33*, *34*). In this study, we substantially expand the application of dCAR T technology to solid tumors and uncover a previously unreported mechanism by which dCAR T cells trigger robust AS, ultimately enabling the systemic eradication of antigen-negative subclones and even abscopal lesions.

In this study, we show that tumor cells treated with dCAR T cells, unlike those exposed to conventional CAR T cells, undergo pronounced ICD, as evidenced by membrane permeabilization, the release of DAMPs (ATP and calreticulin), and increased MHC-I expression. These changes were observed in antigen-negative tumor cells that were never directly recognized by CAR T cells, indicating a nonautonomous, bystander effect likely propagated by cytokine signaling. To date, there is limited evidence demonstrating that CAR T cell therapy itself induces the production of therapeutically potential AS. The priming of DCs has been established as a critical prerequisite for AS in T cells, which is a fundamental process required to induce endogenous T cell immunity (*12*). Recent studies have demonstrated that engineering CAR T cells to secrete cytokines not natively produced by T cells such as IL-12, IL-18, or IL-7, or to constitutively express immunomodulatory molecules like CD40L or CCL19, can potentiate endogenous antitumor immunity (*14*, *16*, *35*, *36*). Although these approaches improved DC activation and promoted endogenous T cell infiltration into the TME, it did not induce ICD in the heterogeneous tumor. Eliciting a durable and specific antitumor response through DC activation alone, without a concomitant increase in target antigen availability, remains challenging. We show that IFN-γ, secreted specifically from dCAR T cells, is the trigger that initiates ICD in antigen-negative tumor cells, in turn activating DCs and priming endogenous T cells.

While both endogenous T cells and CAR T cells in TME are capable of producing IFN-γ, our in vivo studies using *Ifng*^−/−^ dCAR T cells demonstrated that IFN-γ derived specifically from dCAR T cells is essential for initiating AS. Conventional CAR T therapy frequently fails to achieve sufficient intratumoral IFN-γ concentrations to exert tumoricidal effects. Notably, Ma *et al.* (*7*) illustrated that combining CAR T cells with vaccines and STING agonists can enhance IFN-γ production to trigger AS. To ascertain the intrinsic capacity of dCAR T cells to produce high-level IFN-γ without pharmacological intervention, we performed ATAC-seq on resting preinfusion dCAR T cells. Epigenetic modifications by DNA demethylation agents or DNMT3A deletion can affect the expression of transcription factors (*20*, *21*). Consistent with these phenomena, ATAC-seq results revealed heightened chromatin accessibility at IFN-γ–related genes, their regulatory elements, and response genes compared to conventional CAR T cells. Consistently, single-cell transcriptomic analysis of dCAR T cells demonstrated substantially up-regulated expression of IFN-γ key regulators, such as *Tbx21*, *Atf2*, *Nfat*, and *Stat3* (*28*, *29*), as well as the up-regulation of *Gapdh*, as a posttranscriptional modulator that binds to the 3′ untranslated region of IFN-γ to regulate its posttranscriptional expression (*37*). Collectively, these lines of evidence establish that dCAR T cells are epigenetically primed for robust IFN-γ production.

While IFN-γ is a well-characterized cytokine involved in T cell–mediated responses (*7*, *38*, *39*), our study lies in defining a self-reinforcing, one-stop platform by which epigenetically programmed CAR T cells, without further genetic engineering or combination regimens, can intrinsically produce supraphysiological levels of IFN-γ, thereby inducing ICD in antigen-negative tumors, activating DCs, and subsequently initiating a de novo polyclonal endogenous T cell response.

Although the ability of IFN-γ to mediate tumor cell death has been previously established (*30*–*32*), our study reveals how epigenetically programmed CAR T cells can autonomously orchestrate the entire cascade of AS, thereby enabling effective control of antigen-negative and abscopal tumor lesions. By using multiple tumor models, distinct antigen configurations (both surrogate and TAAs), and a suite of functional assays, including ELISPOT, scTCR-seq, DC depletion, IFN-γ neutralization, and *Ifng*^−/−^ dCAR T cells, we have constructed a comprehensive causal framework demonstrating that dCAR T cell therapy is both necessary and sufficient to initiate AS-driven tumor clearance.

In summary, this study demonstrated a potential mechanism for AS generation after dCAR T treatment in that antigen-activated dCAR T cells released substantial amounts of IFN-γ to drive widespread ICD in tumor cells, thereby activating DCs and stimulating endogenous CD8^+^ T cells (Fig. 6H). These insights strongly supported the antitumor function of dCAR T cells in both target antigen-positive and antigen-negative mixed solid tumors and abscopal antigen-negative tumors. Our findings underscore the pivotal role of dCAR T cells in the effective treatment of heterogeneous tumors, and even abscopal heterogeneous tumors, to assert their potential in clinical trials.

## MATERIALS AND METHODS

### Study design

This study was designed to investigate the role of dCAR T cells in inducing AS within endogenous T cells for the eradication of antigenically heterogeneous solid tumors in immunocompetent mice. We generated murine dCAR T cells by transducing murine splenic T cells with a retrovirus, incorporating a 10 nM DAC as described in our previous study (*19*). The antitumor efficacy and TME remodeling capability of dCAR T cells were evaluating using flow cytometry, mass cytometry (CyTOF), and mouse allograft studies. We further analyzed heterogeneous tumor clearance mediated by dCAR T–triggered AS in endogenous T cells through scRNA-seq, ELISPOT, flow cytometry, CD3 antibody blockade, CAR target antigen-positive and antigen-negative mixed tumors and abscopal antigen-negative tumor engraft studies, and antigen-negative tumor rechallenge studies. Last, we elucidated the mechanism of AS within endogenous T cells induced by dCAR T cells through in vitro coculture using a Transwell, flow cytometry, multiomic analyses, and various tumor engraftment studies. For in vivo experiments, C57BL/6, OT-I, and *Batf3*^−/−^ mice aged 6 to 8 weeks were randomly assigned to different treatment groups. During the data collection and analysis, researchers were not blinded to group allocation.

### Cell lines

Cell lines, including OVA^+^MC38, MC38, OVA^+^B16F10, B16F10, and B16F10 expressing luciferase, mCherry, and human CD19 (generated in this study), and MC38 expressing human CD19 (generated in this study), were maintained in completed RPMI 1640 (Gibco), which contained 10% (v/v) fetal bovine serum (FBS; Gibco), penicillin (100 U/ml), and streptomycin (100 mg/ml). 293T cells (American Type Culture Collection) were cultured in Dulbecco’s modified Eagle’s medium (Gibco) supplemented with 10% (v/v) FBS (Gibco), penicillin (100 U/ml), and streptomycin (100 mg/ml).

### Animals

Six- to eight-week-old C57BL/6 mice were purchased from SPF Biotechnology Co., Ltd. Six- to eight-week-old congenic CD45.1^+^ C57BL/6 mice were purchased from Peking University Health Science Center. OT-I and *Batf3*^−/−^ mice were obtained from the Jackson Laboratory. *Ifng*^−/−^ mice were donated by M. Jing (Chinese Institute for Brain Research, Beijing, China). These mice were fed in the animal facility of the Chinese PLA General Hospital under pathogen-free conditions. All animal experiments were administered followed a protocol approved by the PLA Institutional Animal Care and Use Committee (SQ2021216).

### CAR construct and CAR T cell generation

The human CD19-binding single-chain fragment variable (ScFv) sequence was derived from the FMC63 monoclonal antibody. The murine CAR constructs harbored an anti-human CD19 ScFv, a murine CD8a hinge and transmembrane domain, and the murine intracellular domains of 4-1BB and CD3ζ; green fluorescent protein (GFP), which was separated by a self-cleaving 2A peptide sequence (T2A), was generated and cloned into an murine stem cell virus retroviral vector as previous described (fig. S1A) (*40*). The CAR and pCL-ECO plasmid were transfected into 293T cells using the Lipofectamine 3000 transfection reagent. Retroviral supernatants were harvested and stored at −80°C.

Murine lymphocytes were isolated from spleen by using the murine lymphocyte separation kit (Solarbio, China). Lymphocytes were plated at 3 × 10^6^ per well in 24-well plates in RPMI 1640 medium and stimulated with anti-CD3 (500 μg/ml) and anti-CD28 (500 μg/ml) antibodies (BioLegend) and recombinant human IL-2 (rhIL-2; 300 IU/ml; PeproTech). Twenty-four to 48 hours after cell activation, retroviral supernatants were added in retronectin (Takara)–precoated 24-well plates and spun at 2000*g* for 1.5 hours at 32°C. Then, the supernatants were discharged. A total of 1.5 × 10^6^ 24-hour activated cells were added into each well, and polybrene (8 μg/ml; Sigma-Aldrich) was likewise added into each well. In addition, 10 nM DAC (Sigma-Aldrich) was added to the infected system for the preparation of DAC-priming CAR T cells. After centrifugation at 800*g* for 10 min at 32°C, the plates were incubated at 37°C in 5% CO_2_. The retrovirus transduced cells were transferred to new plates on the following day, and further expanded with rhIL-2 (300 IU/ml).

### Flow cytometric analysis

The immunophenotype of the dCAR T cell products was assessed using flow cytometry with fluorescently labeled anti-mouse antibodies specific for CD3, CD4, CD8, CD44, CD62L, TIM3, LAG3, and PD1 (BioLegend). dCAR T cells were collected after a total of 10 days in culture and stained with these antibodies for 30 min at 4°C in the dark. Control staining was accomplished by isotype-matched monoclonal antibodies. Expression of GFP on T cells was detected to determine the transduction efficiency.

For a degranulation assay, dCAR T-19 cells were cocultured with CD19^+^B16 cells at an effector:target (E:T) ratio of 1:1 containing an anti-mouse CD107a antibody (BioLegend) for 1 hour, followed by incubation with a Golgi Plug protein transport inhibitor (BD Biosciences) for 3 hours. For an intracellular cytokine assay, dCAR T-19 cells were cocultured with CD19^+^B16 cells at an E:T ratio of 1:1 containing a Golgi Plug protein transport inhibitor for 4 hours. After that, cells were fixed/permeabilized by using the Foxp3/Transcription Factor Staining Buffer Set (Invitrogen) for 1 hour at 4°C and washed with permeabilization buffer. Then, cells were stained with anti-mouse IL-2, IFN-γ, TNF-α, perforin, and granzyme B antibodies (BioLegend) at room temperature for 30 min, and again washed with permeabilization buffer.

All samples were assessed using a DxFLEX flow cytometry (BECKMAN COULTER). Data were analyzed with Kaluza analysis 2.0 (BECKMAN COULTER) and FlowJo software v.10 (FlowJo LLC).

### Luciferase-based cytotoxicity analysis

CD19^+^B16-luciferase cell-based cytotoxicity was tested as previously described (*41*). CAR T-19 or dCAR T-19 cells were planted in a white opaque plate; then, CD19^+^B16-luciferase cells were added into each well at E:T ratios of 1:1, 1:5, 1:10, 1:20, and 1:30, and the plates were incubated at 37°C in 5% CO_2_ for the indicated times. After that, 100 μl of 2× D-Luciferin solution (300 μg/ml) was added to each well, and luminescence was assessed after 5 min by Varioskan LUX (Thermo Fisher). The percentage of tumor lysis was analyzed by the following formula: 1 − [(value of sample) − (value of negative control)]/[(value of positive control) − (value of negative control)].

### Tumor cell death

For tumor cell death under IFN-γ stimulation, tumor cells (5 × 10^4^ cells per well) were seeded in 12-well tissue culture plates and were stimulated with recombinant mouse IFN-γ (20 ng/ml; PeproTech) for 48 hours. After that, cell death was determined by an apoptosis detection kit following the manufacturer’s protocol.

For the membrane permeabilization of antigen-positive tumor cells, tumor cells were cocultured with carboxyfluorescein succinimidyl ester prestained dCAR T-19 or CAR T-19 cells at an E:T ratio of 1:1 for 24 hours. Then, an apoptosis detection kit (BD Biosciences) was used to analyze the membrane permeabilization of tumor cells.

For the membrane permeabilization of antigen-negative tumor cells, a Transwell was used in this in vitro experiment. In the upper inserts, antigen-positive tumor cells were cocultured with dCAR T-19 or CAR T-19 cells at an E:T ratio of 1:1, and antigen-negative tumor cells were seeded in the lower wells. After culturing for 24 hours, antigen-negative tumor cells in the lower well were analyzed by an apoptosis detection kit.

### Animal experiments

Six- to eight-week-old C57BL/6 mice were subcutaneously injected with 3 × 10^5^ CD19^+^B16 tumor cells. Mice were subsequently lymphodepleted by intraperitoneal injections of 200 μg of cyclophosphamide and 100 μg of fludarabine after 4 and 5 days, respectively. Seven days later, mice were randomly divided into three groups and received tail intravenous injection with 1 × 10^7^ untransduced T, CAR T-19, and dCAR T-19 cells per mouse. For tumor rechallenged experiments, CD19^+^B16 tumor cells were subcutaneously injected on the opposite flank of the surviving mice that achieved complete response, and tumor-naïve and age-matched C57BL/6 mice were used as control.

For AS trigged by dCAR T experiments, 6- to 8-week-old CD45.2^+^ wild-type, *Batf3*^−/−^ C57BL/6 mice were subcutaneously injected with 3 × 10^5^ premixed at a ratio of 1:1 CD19^+^B16:OVA^+^B16 or CD19^+^MC38:OVA^+^MC38 cells in the left flank and 1.5 × 10^5^ OVA^+^B16 or OVA^+^MC38 cells in the right flank. Subsequently, 1 × 10^7^ congenic CD45.1^+^ CAR T-19 and dCAR T-19 cells were intravenously transfer into recipients via the tail vein.

For AS trigged by TCR T cell experiments, 6- to 8-week-old CD45.1^+^ C57BL/6 mice were subcutaneously injected with 3 × 10^5^ premixed at a ratio of 1:1 OVA^+^B16:OVA^−^B16 cells in the left flank and 1.5 × 10^5^ OVA^−^B16 in the right flank. Subsequently, 5 × 10^5^ congenic CD45.2^+^ CD8^+^ OT-1 T and DAC-priming CD8^+^ OT-1 T cells were intravenously transfer into recipients via the tail vein.

For CD3 antibody blockade experiments, 6- to 8-week-old C57BL/6 mice were subcutaneously injected with 3 × 10^5^ premixed at a ratio of 1:1 CD19^+^B16:OVA^+^B16 cells in the left flank and 1.5 × 10^5^ OVA^+^B16 cells in the right flank. Mice were subsequently intraperitoneally injected with 200 μg/mouse anti-mouse CD3 antibodies (BioXcell) after 5 and 6 days, respectively. Seven days later, mice received tail intravenous injection with 1 × 10^7^ CAR T-19 and dCAR T-19 cells per mouse.

For IFN-γ antibody blockade experiments, 6- to 8-week-old C57BL/6 mice were subcutaneously injected with 3 × 10^5^ premixed at a ratio of 1:1 CD19^+^B16: OVA^+^B16 cells in the left flank and 1.5 × 10^5^ OVA^+^B16 cells in the right flank. Seven days later, mice received tail intravenous injection with 1 × 10^7^ CAR T-19 and dCAR T-19 cells per mouse. Mice were subsequently intraperitoneally injected with 500 μg of anti-mouse IFN-γ antibodies (Bioxcell) per mouse 2, 3, and 5 days after ACT.

Tumor size was monitored on the indicated times, and tumor size was analyzed by the following formula: (length×width^2^)/2. Blood, spleen, and tumor samples were collected on the indicated times. Tumor-engrafted mice were euthanized when they showed paralysis, impaired mobility, poor physical condition, or tumors with a diameter greater than 2000 mm^3^.

### CyTOF

Following lymphodepletion, C57BL/6 mice (CD45.2^+^) bearing CD19^+^B16 tumor cells were injected with 1 × 10^7^ congenic CD45.1^+^ CAR T-19 and dCAR T-19 cells. We collected tumor tissues from five mice per group on day 7 after ACT. Each group’s tissues were pooled and dissociated into single cells using a Tissue Dissociation Kit. Next, CD45^+^ cells were then isolated using magnetic beads for CyTOF assay.

Cisplatin-194Pt was added into CD45^+^ cell suspensions and incubated at 4°C for 5 min. Next, cells were washed with PBS twice and incubated with block mix at 4°C for 20 min. After that, cells were stained with the desired metal-conjugated surface antibody cocktails for immune cells, including CD4^+^ CAR T (CD45.1^+^CD3^+^CD4^+^), CD8^+^ CAR T (CD45.1^+^CD3^+^CD8^+^), endogenous CD4^+^ T (CD45.2^+^CD3^+^CD4^+^), endogenous CD8^+^ T (CD45.2^+^CD3^+^CD8^+^), NK (CD45.2^+^CD3^−^CD49b^+^CD161^+^), DCs (CD45.2^+^CD11b^+^MHC-II^+^CD11c^+^CD86^+^), and macrophage (CD45.2^+^CD11b^+^F4/80^+^CD86^−^CD11c^−^), and they were incubated at 4°C for 30 min. Cells were stained with iridium-191/193 after being fixed and permeabilized with Foxp3/transcription factor staining buffer at 4°C overnight. Subsequently, cells were sequentially stained with a metal-conjugated intracellular antibody mix at 4°C for 30 min. A CyTOF Helios mass cytometer (Fluidigm) was used to analyze and acquire data, which normalized to the EQ bead signal, debarcoded using a doublet filtering scheme, and analyzed with FlowJo software v.10.

### ELISPOT

Endogenous T cells were isolated from spleen to evaluate AS by measuring their IFN-γ expressing as previously described (*7*). First, to acquire endogenous CD45.1^+^ or CD45.2^+^ cells, splenocytes were stained with phycoerythrin (PE)-conjugated anti-mouse CD45.1 or CD45.2 antibodies (BioLegend), and then anti-PE-beads (MACS) were added and incubated. Next, CD45.1^+^ or CD45.2^+^ cells were collected from effluent by using magnetic cell separation. After that, CD3^+^ T cells were isolated from CD45.1^+^ or CD45.2^+^ cells by using a CD3^+^ T cell isolation kit (BioLegend).

A total of 2 × 10^5^ CD3^+^ T cells were mixed with 2 × 10^4^ cells pretreated with 100 IU of murine IFN-γ and irradiated cancer cells in 200 μl of complete medium and seeded in a 96-well ELISPOT plate precoated with IFN-γ capture antibody (BD Biosciences). After incubation for 24 hours at 37°C in the dark, IFN-γ production was assessed with an IFN-γ ELISPOT Assay Kit (BD Biosciences) according to the manufacturer’s protocol. Then, an ImmunoSpot S6 Analyser (Cellular Technology Limited) was used to enumerate cytokine spots.

### ELISA

Cytokine measurement in culture supernatant: dCAR T-19 cells were cocultured with CD19^+^B16 cells at an E:T ratio of 1:1 in 1 ml of completed RPMI 1640 medium for 24 hours. After that, supernatants were collected and IL-2, IFN-γ, and TNF-α production in the supernatants was quantified by an enzyme-linked immunosorbent assay (ELISA) kit (Novus) according to the manufacturer’s protocol.

Cytokine measurement in serum: Blood was obtained in heparin-coated tubes via tail vein bleed, and then serum was collected after blood spun at 2000 rpm for 10 min at 4°C and stored at −80°C. IFN-γ, calreticulin, high mobility group box 1 (HMGB1) (Novus), and ATP (Absin) levels were determined by an ELISA kit according to the manufacturer’s protocol.

Cytokine measurement in tumor: Tumors were collected on day 7 after tumor implantation. Then, 100 mg of tumors was applied to the homogenate in 100 μl of PBS. The tumor samples were centrifuged at 2000 rpm for 10 min at 4°C, and the supernatants were collected. IFN-γ, ATP, calreticulin, and HMGB1 levels were determined by ELISA kit according to the manufacturer’s protocol.

### Phenotyping of immune cells in spleens and tumors

Spleens were collected and dissociated in 1 ml of PBS. Splenocytes were collected after being spun at 2000 rpm for 10 min at 4°C. The tumors from mice receiving CAR T or dCAR T cell therapy were surgically collected and weighed. Then, the tumors were dissociated into single-cell suspension by the Tissue Dissociation Kit (MACS) according to the manufacturer’s protocol, and single cells were collected by passing tumors through a 70 mM cell strainer and resuspended with 100 μl of PBS per 200 mg of tumor.

Immunophenotyping analysis on splenocytes: Splenocytes were stained with CD3 and CD45 antibody cocktail and then the expression of GFP on CD3^+^ T cells was detected to determine the CAR^+^ T cells in spleens.

Immunophenotyping analysis on immune cells in tumors: 50 μl of the preprepared tumor cell suspension was washed and stained with the desired antibody cocktails for host immune cells, including T (CD45.2^+^CD3^+^), NK (CD45.2^+^CD3^−^NK1.1^+^), CD11b (CD45.2^+^CD11b^+^), DCs (CD45.2^+^Ly6G^−^CD11b^−^CD11c^+^), and M2 (CD45.2^+^Ly6G^−^CD11b^+^F4/80^+^CD206^+^) (*42*), as well as CD103^+^DCs (CD45.2^+^Ly6c^−^MHC II^+^F4/80^−^CD11b^−^CD103^+^) (*15*, *43*), for 30 min at 4°C and followed by twice washing with 1 ml of PBS. After that, cells were resuspended in PBS for flow cytometry analysis.

Tetramer staining on immune cells in tumors: 50 μl of the preprepared tumor cell suspension was washed and stained with 10 μl of PE conjugate-SIINFEKL-Tetramer or PE conjugate-KSPWFTTL-Tetramer (MBL) plus Fc block for 60 min at 4°C in the dark, followed by staining with a premixed antibody cocktail for endogenous OVA-specific CD8^+^ T cells (CD45.2^+^CD3^+^CD8^+^SIINFEKL-Tetramer^+^) or MuLV p15E-specific CD8^+^ T cells (CD45.2^+^CD3^+^CD8^+^ KSPWFTTL-Tetramer^+^) for 30 min at 4°C in the dark. Cells were then washed twice and analyzed with flow cytometry.

### ATAC sequencing

CAR^+^ T cells were sorted by flow cytometry after 10 days of CAR T and dCAR T cells cultured in vitro. Nuclei were isolated from approximately 1 × 10^5^ sorted CAR^+^ T cells using ATAC-LB. Samples were submitted to LC-Bio for library construction and sequencing. Then, a transposition reaction with Tn5 transposase (Illumina) was performed for 30 min at 37°C with 1000 rpm mixing. The DNA was purified using the MinElute PCR Purification Kit (Qiagen) after tagmentation. After that, the tagmented DNA was amplified with Phusion high-fidelity PCR master mix (NEB) for 14 PCR cycles. The amplified libraries were further purified with the MinElute PCR Purification Kit. Before sequencing, a double-size selection of the final libraries was made using AMPure beads. Paired-end sequencing (2 × 75 bp reads) was performed on an Illumina NextSeq 5000 platform.

### ATAC-seq analysis

The quality of raw sequences was analyzed by using FASTQ software, while Cutadapt filters and extracts clean reads of high quality. The sequencing data are aligned to reference genomes using bwa-mem2. Reads with an MAPQ score of 30 or higher are selected for comparison, and duplicate reads are excluded. After alignment, the results by genome alignment positions are sorted by using Samtools, and the duplicate alignments are removed by using rmdup. Bedtools then converts the bam file into a bed file for further analysis.

MACS2 software was used to identify and assess regions of the genome with high read length abundance. Set the threshold *P* value to <0.001 to select significant peaks from the initial results. The Homer software was used to annotate all peaks, and the peaks within 500 bp of the gene were designated as ATAC-seq peaks for subsequent analysis.

The log2-fold change in peak intensity between two samples was calculated using DESeq2, and a *q* value < 0.1 was applied as the threshold for the difference peak. After that, functional enrichment analyses were performed using KOBAS software, focusing on the GO and KEGG pathways of genes affected by the identified high-confidence differential peaks.

The peaks located within 5 kb upstream and downstream of annotated genes were used as inputs for motif detection using Dreme software. The detected motifs are then compared with known motifs in the motifs database (http://meme-suite.org/db/motifs) using TOMTOM software. Entries with *q* value < 0.05 were considered as significant results.

### Single-cell RNA sequencing

C57BL/6 mice (CD45.2^+^) bearing 3 × 10^5^ premixed at a ratio of 1:1 CD19^+^B16: OVA^+^B16 cells were treated with congenic CD45.1^+^ CAR T-19 and dCAR T-19 cells; tumor tissues were collected and dissociated into single cells by using the Tissue Dissociation Kit 7 days after ACT. Cell suspension was pelleted and total RNA was extracted by using the RNeasy Mini Kit (QIAGEN) following the manufacturer’s protocol. Sequencing libraries were prepared using a chromium Next GEM Single Cell 3′ v3.1 kit (10× Genomics) according to the manufacturer’s protocol. Index demultiplexing and FASTQ generation were performed by LC-Bio. Raw gene expression matrices were generated using the Cell Ranger (version 6.1.1) pipeline in combination with the mm10 genome annotation reference. Cells expressing between 500 and 5000 detected genes or with a mitochondrial gene expression below 25% were retained. Seurat software package v4.1.1 in R was used to filter out fragments and dead cells, and Seurat was used for clustering of the scRNA-seq profile for dimensional-reduction UMAP and differential gene analysis.

### Single-cell TCR-sequencing data analysis

RNA samples from tumor-dissociated single cells collected from C57BL/6 mice (CD45.2^+^) bearing 3 × 10^5^ premixed at a ratio of 1:1 CD19^+^B16: OVA^+^B16 cells after treatment with congenic CD45.1^+^ CAR T-19 and dCAR T-19 cells were used for analysis. According to the manufacturer’s protocol, the libraries for single-cell TCR sequencing were prepared using 10× Genomics 5′ Single Cell Immune Profiling technology, and TCR clonotype identification, alignment, and annotation were performed using the 10× Genomics Cell Ranger pipeline (version 6.1.1) as previously described (*44*). Clonotype alignment was performed against the Cell Ranger mouse V(D)J reference library 3.1.0 (GRCm39 and Ensembl GTF version 94). Define the TCR clone in scRepertoire using the “combineTCR” function (*45*). Clonal amplification was analyzed using a four-group classification. The percentage of cells in various clones was assessed for endogenous CD8^+^ T cells in TILs.

### scRNA-seq cluster annotation

Cell type–specific gene signature lists derived from multiple references were used to annotate different clusters for scRNA-seq analysis (*7*, *25*, *46*, *47*). Cell type–specific gene signature lists were used as follows: cancer cells: *Pmel*, *Mlana*, *Tyrp1*, and *Dct*; T/NK cells: *Cd3d*, *Cd3e*, *Cd3g*, and *Nkg7*; B cells: *Cd79a*, *Cd79b*, and *Cd19*; myeloid cells: *Ly6a*, *Med21*, *Itgam*, Cd68, *Cxcl2*, and *Lyz2*; fibroblasts: *Dcn*, *Col1a1*, and *Col1a2*; endothelial cells: *Pecam1*; T cells: *Cd3d*, *Cd3e*, and *Cd3g*; DCs: *Itgax*, *Cst3*, and *Cd74*; conventional DC1 (cDC1): *Itgae*, *Clec9a*, *Ccl17*, *Rf8*, *Wdfy4*, and *Xcr1*; cDC2: *Fcer1a* and *Clec10a*; migration DC (migDC): *Ccr7*, *Ccl22*, and *Fscn1*; plasmacytoid DC (pDC): *Bst2*, *Ly6d*, *Siglech*, and *Tcf4*. Human CD19-expression tumor cells were annotated by human CD19 gene sequence and CAR^+^ T cells were annotated by GFP gene sequence, both of which were included in table S1.

### Distributed validation of tumor-infiltrating endogenous CD4^+^ effector T cells

To analyze the distribution of endogenous CD4^+^ effector T cells in tumors treated with CAR T or dCAR T cells, the expression levels of *Ifng*, *Tnf*, and *Ccl5*, markers of effector T cells, were determined in scRNA-seq data according to a previous study (*48*). *y* = log_2_ (*x* + 1) was used to quantify the gene expression, where the average expression level was used to indicate *x*. The relative content of endogenous CD4^+^ effector T cells was analyzed using the following formula: [(*y_Ifng_* + *y_Tnf_* + *y_Ccl5_* + *y_Cd4_*)/4]/[(*y_Cd3d_* + *y_Cd3e_* + *y_Cd3g_*)/3].

### Statistics

Sample size was not predetermined using any statistical method. No data were excluded from the analyses. Tumor-bearing mice were randomly assigned to ACT treatment groups. Statistical analysis was performed with Prism 8.0 (GraphPad). The data points represent bioreplicates and are presented as mean ± SD, where the central values and error bars for tumor growth curves are expressed as mean ± SEM. The two-tailed Student’s *t* test was used to conduct statistical comparisons between two groups, and a two-tailed *P* value < 0.05 was considered significant. Outcomes across multiple groups were determined by analysis of variance (ANOVA) models. The log-rank (Mantel-Cox) test was performed to compare survival curves among groups. Statistical significance is denoted as follows: *P < 0.05; **P < 0.01; ***P < 0.001. All statistical tests and correction methods are detailedly stated in the figure legends.
