# Supplementary material for: Antigen spreading mediates heterogeneous solid tumor eradication by DNA demethylating agent–programmed CAR T cells
Source: Sci Adv. 2026 May 8;12(19):eadz4088. doi: 10.1126/sciadv.adz4088 (PMC13155291; doi:10.1126/sciadv.adz4088)
Supplement: Supplementary file 1 — Figs. S1 to S15 Table S1 [file sciadv.adz4088_sm.pdf]

Science Advances
AAAS

Supplementary Materials for

Antigen spreading mediates heterogeneous solid tumor eradication by DNA demethylating agent–programmed CAR T cells

Yelei Guo *et al.*

Corresponding author: Yao Wang, wangyao_301@hotmail.com; Weidong Han, hanwdrsw@163.com

Sci. Adv. **12**, eadz4088 (2026)
DOI: 10.1126/sciadv.adz4088

This PDF file includes:

Figs. S1 to S15
Table S1

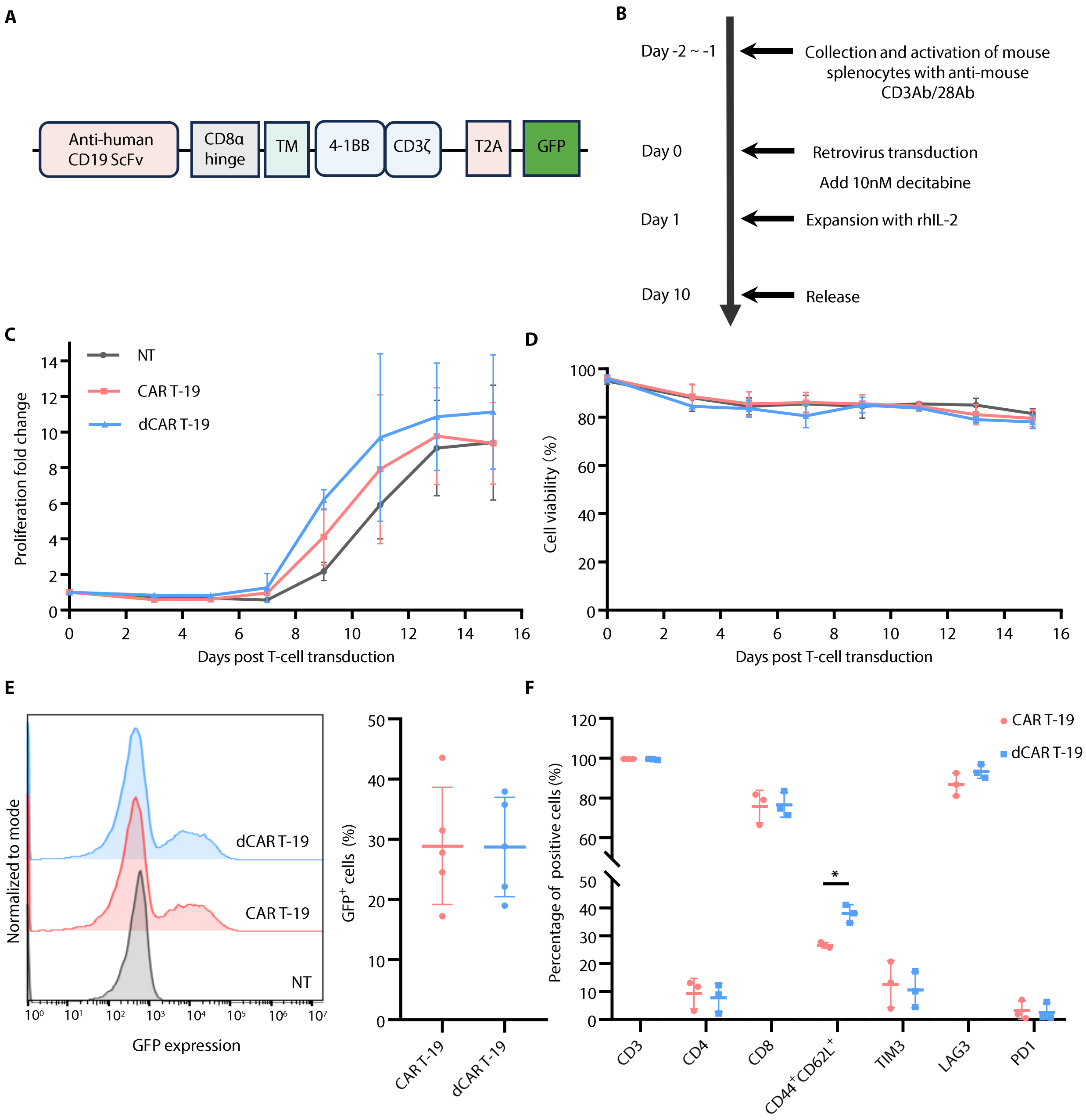

A
Anti-human CD19 ScFv
CD8α hinge
TM
4-1BB
CD3ζ
T2A
GFP
B
Day -2 ~ -1
Collection and activation of mouse splenocytes with anti-mouse CD3Ab/28Ab
Day 0
Retrovirus transduction
Add 10nM decitabine
Day 1
Expansion with rhIL-2
Day 10
Release
C
NT
CAR T-19
dCAR T-19
Proliferation fold change
Days post T-cell transduction
D
Cell viability (%)
Days post T-cell transduction
E
dCAR T-19
CAR T-19
NT
Normalized to mode
GFP expression
GFP+ cells (%)
CAR T-19
dCAR T-19
F
CAR T-19
dCAR T-19
Percentage of positive cells (%)
*
CD3
CD4
CD8
CD44+CD62L+
TIM3
LAG3
PD1

Fig. S1. Manufacturing of low-dose decitabine primed mouse CAR T cells
(**A**) Schematic representation of anti-human CD19 mouse CAR, not to scale. TM, transmembrane. (**B**) Flowchart for manufacture of low-dose decitabine primed mouse CAR T (dCAR T) cells. rhIL-2, recombinant human IL-2. (**C** and **D**) Proliferation (C) and cell viability (D) for dCAR T-19 compared with CAR T-19 or untransduced T (NT) cells (n = 2). (**E**) Percentage of GFP expression on CAR T and dCAR T cells (n = 5). (**F**) Immunophenotypic analysis of dCAR T cell products (n = 3). Statistical significance was determined by two-way ANOVA (C, D) and two-tailed paired Student's t-test (E, F). *$P < 0.05$.

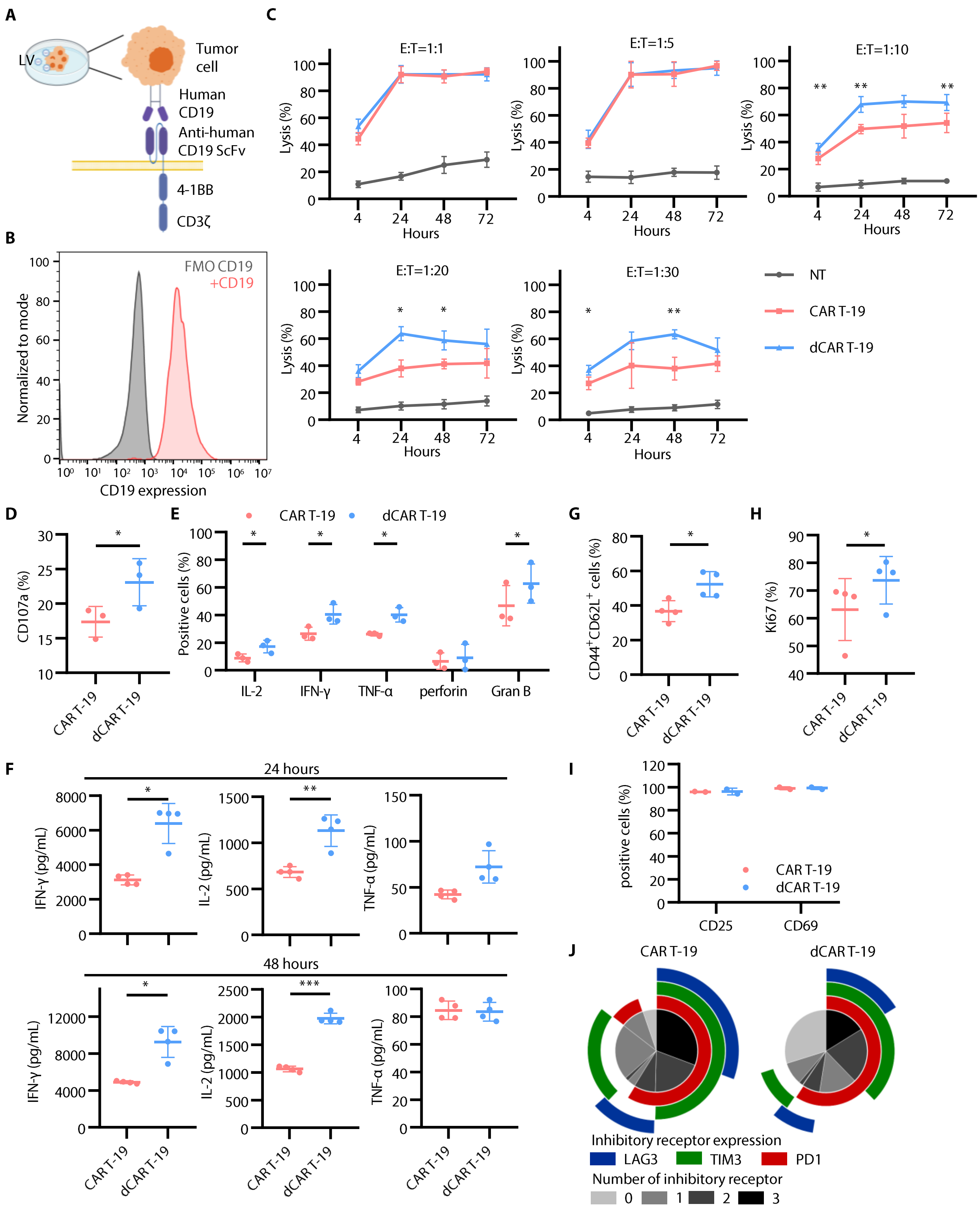

A
LV
Tumor cell
Human CD19
Anti-human CD19 ScFv
4-1BB
CD3ζ
B
FMO CD19
+CD19
Normalized to mode
CD19 expression
C
E:T=1:1
E:T=1:5
E:T=1:10
E:T=1:20
E:T=1:30
Lysis (%)
Hours
NT
CAR T-19
dCAR T-19
D
CD107a (%)
E
Positive cells (%)
IL-2
IFN-γ
TNF-α
perforin
Gran B
G
CD44+CD62L+ cells (%)
H
Ki67 (%)
F
24 hours
48 hours
IFN-γ (pg/mL)
IL-2 (pg/mL)
TNF-α (pg/mL)
I
positive cells (%)
CD25
CD69
J
Inhibitory receptor expression
LAG3
TIM3
PD1
Number of inhibitory receptor
0
1
2
3

Fig. S2. In vitro antitumor ability of dCAR T cells

(**A**) Generation of mouse tumor cells constitutively expressed human CD19 by using lentivirus (LV) transduction. Image created with biogdp.com. (**B**) Representative flow plot for expression of human CD19 on tumor cells. Gates were established using CD19 FMO control. (**C**) Cytotoxic analysis of dCAR T, CAR T and NT cells cocultured with $CD19^{+}$B16 cells at an E:T ratio of 1:1 to 1:30 (n = 4). (**D**) CD107a expression on dCAR T cells compared with CAR T cells after coculture with $CD19^{+}$B16 cells at an E:T ratio of 1:1 for 4h (n = 3). (**E**) Intracellular cytokine expression on dCAR T cells compared with CAR T cells after coculture with $CD19^{+}$B16 cells at an E:T ratio of 1:1 for 4h (n = 3). (**F**) Cytokine production by dCAR T and CAR T cells cocultured with $CD19^{+}$B16 cells at an E:T ratio of 1:1 for 24 and 48 h (n = 4). (**G to J**) CD44/CD62L (n = 4) (G), Ki67 (n = 4) (H), CD25 and CD69 (n = 2) (I), and differences in the ratio of cells expressing 0, 1, 2 or 3 phenotypes (PD1, LAG3 and TIM3) expression (J) on CAR T and dCAR T cells was performed after cocultured with $CD19^{+}$B16 cells at an E:T ratio of 1:1 for 24h. Statistical significance was determined by two-tailed paired Student's t-test (C to I). ***$P < 0.001$; **$P < 0.01$; *$P < 0.05$.

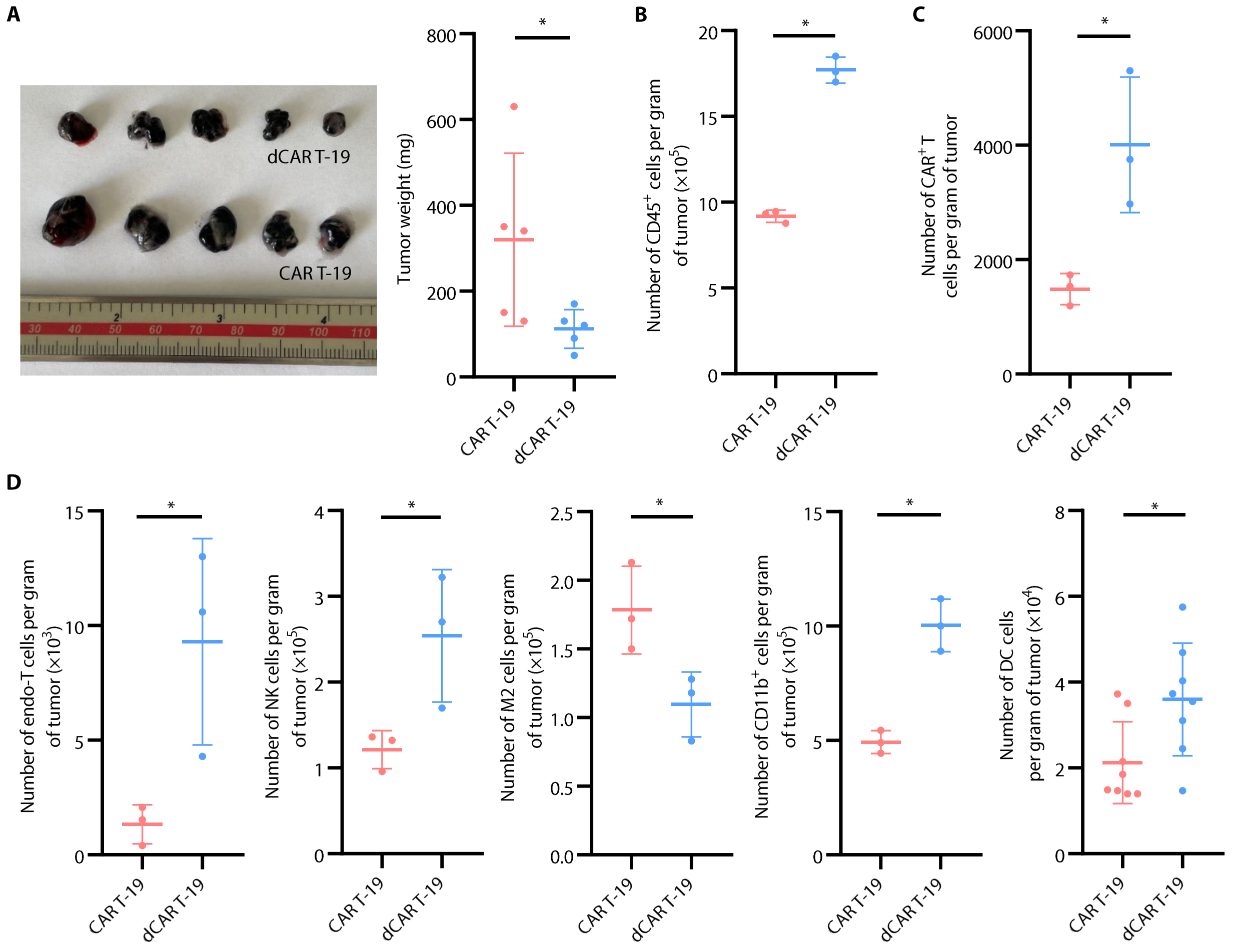

A
dCAR T-19
CAR T-19
Tumor weight (mg)
B
Number of CD45+ cells per gram of tumor (×10^5)
C
Number of CAR+ T cells per gram of tumor
D
Number of endo-T cells per gram of tumor (×10^3)
Number of NK cells per gram of tumor (×10^5)
Number of M2 cells per gram of tumor (×10^5)
Number of CD11b+ cells per gram of tumor (×10^5)
Number of DC cells per gram of tumor (×10^4)
CAR T-19
dCAR T-19
*

Fig. S3. The changes of tumor-infiltrating immune cells following dCAR T cell therapy
(**A**) Tumor photographs (left) and tumor weight (right) in mice attributed from in vivo experiment (Fig. 1E) (n = 5). (**B** to **D**) Flow cytometry analysis on tumor-infiltrating immune cells in mice bearing $CD19^{+}$B16 tumors after ACT for 7 days. (**B**) The number of tumor-infiltrating $CD45^{+}$ cells in mice in each treated-group (n = 3). (**C** and **D**) The number of tumor-infiltrating CAR^{+} T (C) and endogenous immune cells (D) in mice in each treated-group (n = 8 in DC, n = 3 in other cells). Statistical significance was determined by two-tailed paired Student's t-test (A to D). *$P < 0.05$; **$P < 0.01$.

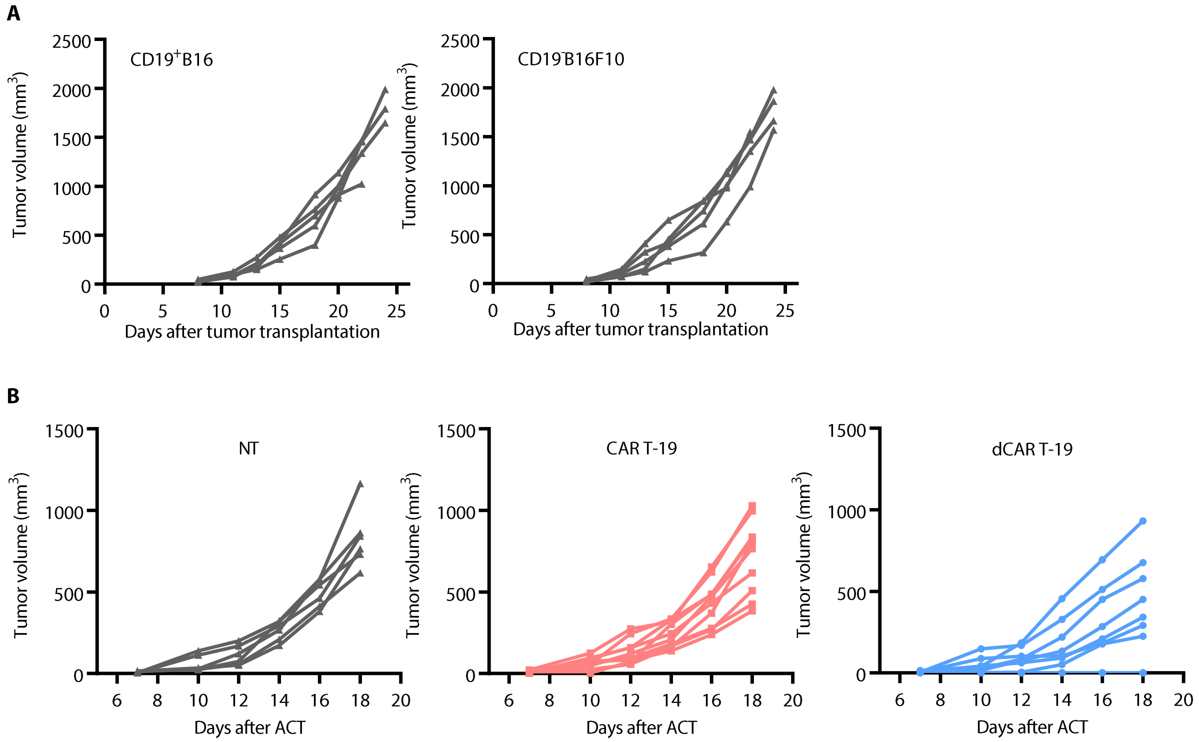

A
CD19+B16
CD19-B16F10
Tumor volume (mm3)
Days after tumor transplantation
B
NT
CAR T-19
dCAR T-19
Tumor volume (mm3)
Days after ACT

Fig. S4. The change of tumor volumes following adoptive cell therapy
(**A**) Tumor growth of $CD19^{+}$B16 and $CD19^{-}$B16F10 cells after subcutaneously transplanted in C57BL/6 mice. (**B**) Individual tumor growth in mice upon ACT attributed from in vivo experiment (Fig. 2A).

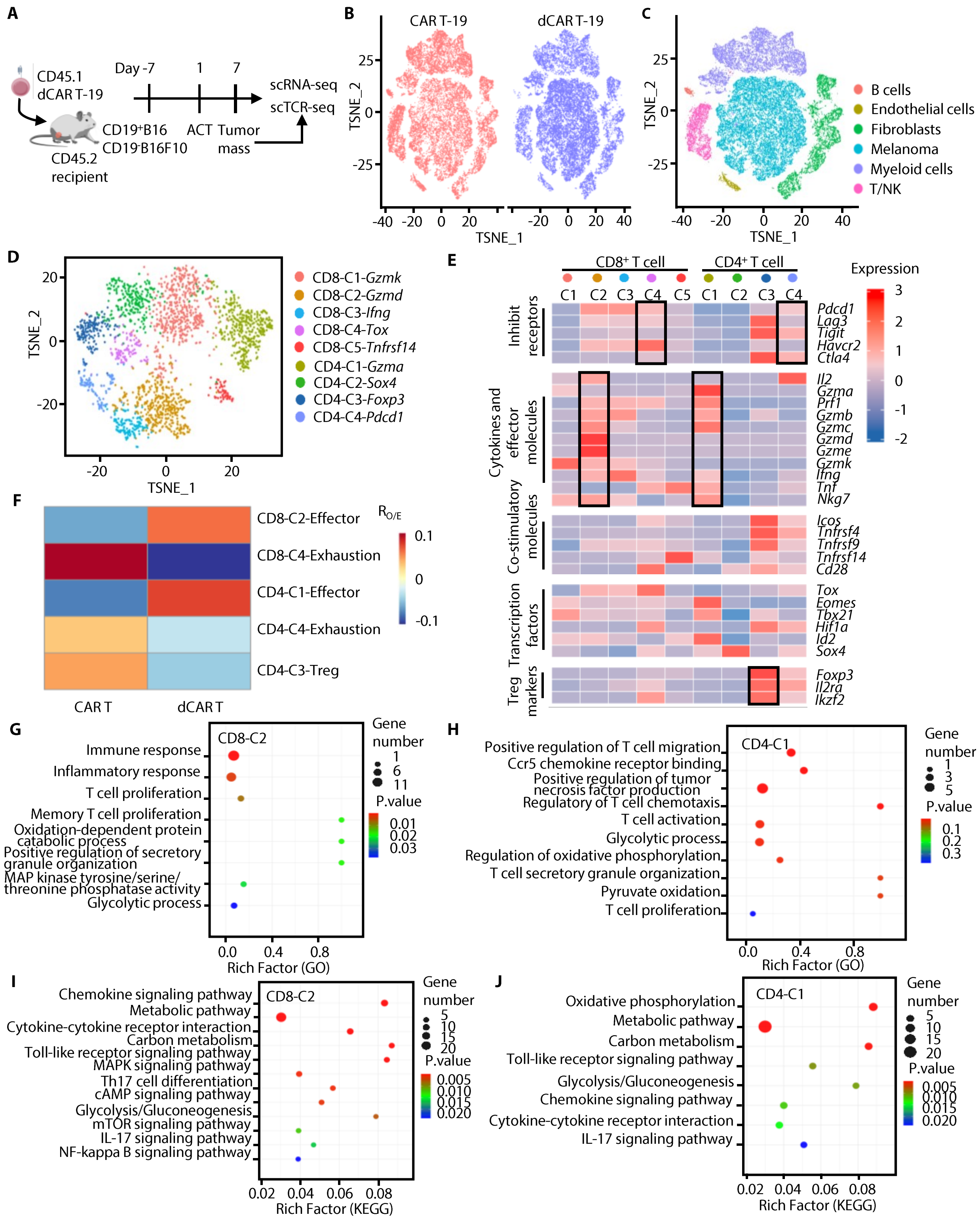
A
CD45.1
dCAR T-19
Day -7
1
7
scRNA-seq
scTCR-seq
CD19+B16
CD19-B16F10
ACT
Tumor mass
CD45.2 recipient
B
CAR T-19
dCAR T-19
TSNE_2
TSNE_1
C
B cells
Endothelial cells
Fibroblasts
Melanoma
Myeloid cells
T/NK
D
CD8-C1-Gzmk
CD8-C2-Gzmd
CD8-C3-Ifng
CD8-C4-Tox
CD8-C5-Tnfrsf14
CD4-C1-Gzma
CD4-C2-Sox4
CD4-C3-Foxp3
CD4-C4-Pdcd1
E
CD8+ T cell
CD4+ T cell
Expression
Inhibit receptors
Cytokines and effector molecules
Co-stimulatory molecules
Transcription factors
Treg markers
Pdcd1
Lag3
Tigit
Havcr2
Ctla4
Il2
Gzma
Prf1
Gzmb
Gzmc
Gzmd
Gzme
Gzmk
Ifng
Tnf
Nkg7
Icos
Tnfrsf4
Tnfrsf9
Tnfrsf14
Cd28
Tox
Eomes
Tbx21
Hif1a
Id2
Sox4
Foxp3
Il2ra
Ikzf2
F
CD8-C2-Effector
CD8-C4-Exhaustion
CD4-C1-Effector
CD4-C4-Exhaustion
CD4-C3-Treg
RO/E
CAR T
dCAR T
G
CD8-C2
Immune response
Inflammatory response
T cell proliferation
Memory T cell proliferation
Oxidation-dependent protein catabolic process
Positive regulation of secretory granule organization
MAP kinase tyrosine/serine/threonine phosphatase activity
Glycolytic process
Gene number
P.value
Rich Factor (GO)
H
CD4-C1
Positive regulation of T cell migration
Ccr5 chemokine receptor binding
Positive regulation of tumor necrosis factor production
Regulatory of T cell chemotaxis
T cell activation
Glycolytic process
Regulation of oxidative phosphorylation
T cell secretory granule organization
Pyruvate oxidation
T cell proliferation
Rich Factor (GO)
I
CD8-C2
Chemokine signaling pathway
Metabolic pathway
Cytokine-cytokine receptor interaction
Carbon metabolism
Toll-like receptor signaling pathway
MAPK signaling pathway
Th17 cell differentiation
cAMP signaling pathway
Glycolysis/Gluconeogenesis
mTOR signaling pathway
IL-17 signaling pathway
NF-kappa B signaling pathway
Rich Factor (KEGG)
J
CD4-C1
Oxidative phosphorylation
Metabolic pathway
Carbon metabolism
Toll-like receptor signaling pathway
Glycolysis/Gluconeogenesis
Chemokine signaling pathway
Cytokine-cytokine receptor interaction
IL-17 signaling pathway
Rich Factor (KEGG)

Fig. S5. Transcriptional changes of tumor-infiltrating endogenous T cells analyzed by scRNA-seq

(**A**) Schematic of in vivo experimental design for B to J. Congenic CD45.1$^+$ CAR T-19 or dCAR T-19 cells were adoptively transfer into C57BL/6 recipients (CD45.2$^+$), which were subcutaneously engrafted with CD19$^+$B16 and CD19$^-$B16F10 mixed tumors. On day 7 after ACT, tumor masses were analyzed by scRNA-seq and scTCR-seq (n = 2). Image created with biogdp.com. (**B**) TSNE views indicating comprehensive scRNA-seq analysis. (**C**) Curated cell clusters based on signature gene expression. (**D**) TSNE views indicating comprehensive single-cell analysis of tumor-infiltrating endogenous T cells, showing the formation of 9 clusters, containing 5 for CD8$^+$ T cels and 4 for CD4$^+$ T cells. (**E**) Mean expression of indicated T cell function-related genes in each cell cluster.

(**F**) Tumor preference of each T cell cluster. The ratio of observed cell numbers to random expectation was used to distinguish cell-sampling biases. (**G** and **H**) Upregulation of GO functional clustering of genes in CD8-C2 (G) and CD4-C1 (H) clusters in dCAR T-treated tumor cells compared with that in CAR T-treat tumor cells. **(I** and **J**) Upregulation KEGG functional clustering of genes in CD8-C2 (I) and CD4-C1 (J) clusters in dCAR T-treated tumor cells compared with that in CAR T-treat tumor cells.

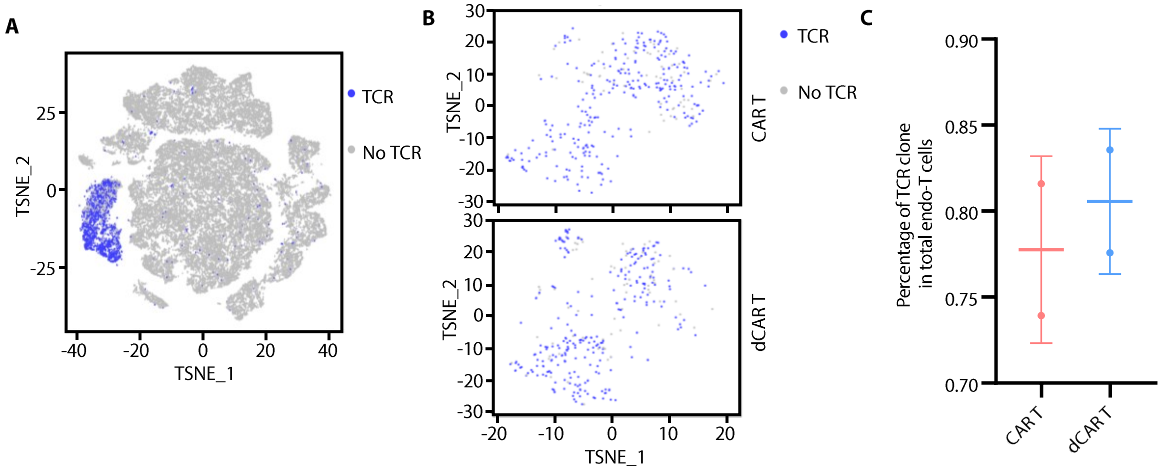
A
TCR
No TCR
TSNE_2
TSNE_1
25
0
-25
-40
-20
0
20
40
B
TCR
No TCR
TSNE_2
TSNE_1
CAR T
dCAR T
30
20
10
0
-10
-20
-30
-20
-10
0
10
20
C
Percentage of TCR clone in total endo-T cells
0.90
0.85
0.80
0.75
0.70
CAR T
dCAR T

Fig. S6. TCR clonotype analysis of endogenous CD8$^+$ T cells

(**A**) Plots showing TSNE views of TCR clonotype on T/NK cluster by comprehensive scRNA-seq and scTCR-seq analysis derived from fig S5A (*n* = 2). (**B**) Plots showing TSNE views of TCR clonotype by comprehensive scRNA-seq and scTCR-seq analysis on endogenous CD8$^+$ T cells (*n* = 2). (**C**) Percentage of TCR clone in total endogenous CD8$^+$ T cells (*n* = 2).

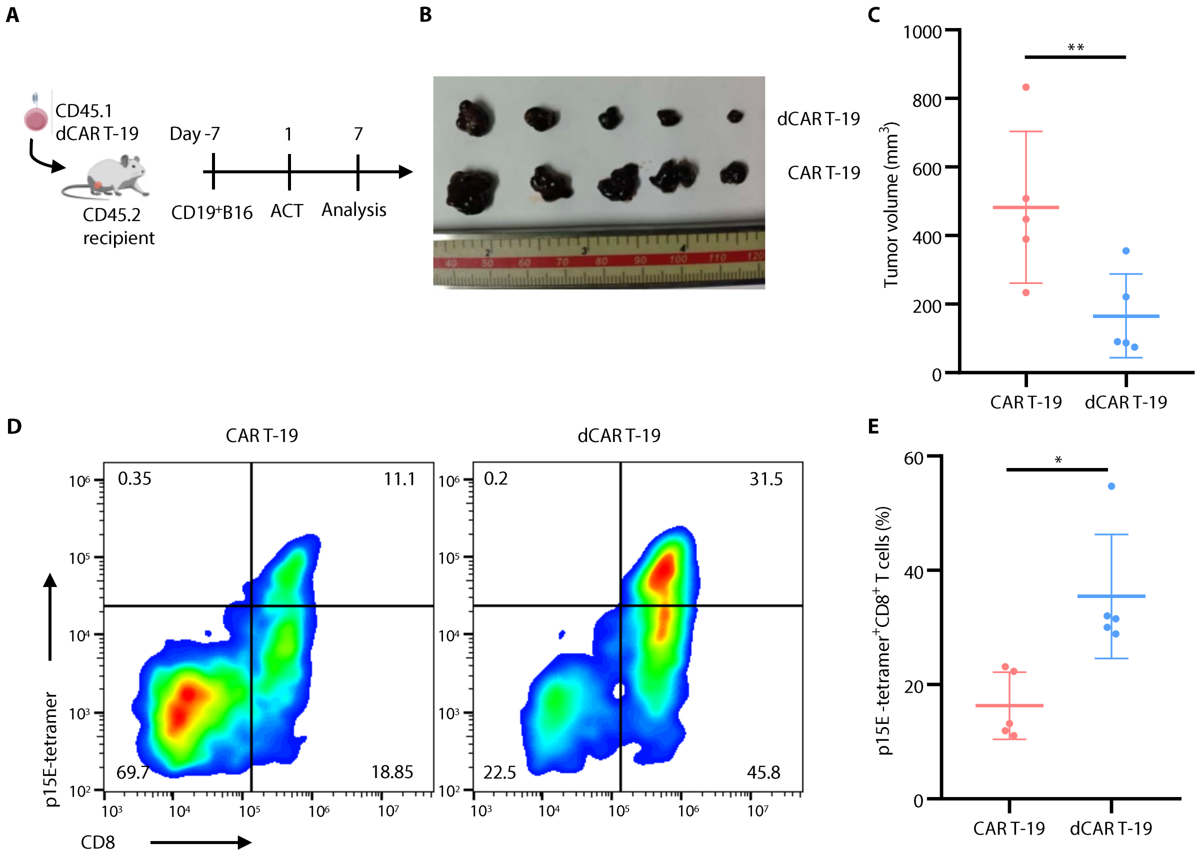
A
CD45.1
dCAR T-19
CD45.2
recipient
Day -7
1
7
CD19⁺B16
ACT
Analysis
B
dCAR T-19
CAR T-19
C
Tumor volume (mm3)
**
CAR T-19
dCAR T-19
D
CAR T-19
dCAR T-19
0.35
11.1
69.7
18.85
0.2
31.5
22.5
45.8
p15E-tetramer
CD8
E
p15E -tetramer+CD8+ T cells (%)
*
CAR T-19
dCAR T-19

fig. S7. dCAR T cells promoted antigen spreading in the $CD19^+$B16 murine model
(**A**) Schematic of in vivo experimental design. C57BL/6 (CD45.2) mice were administered dCAR T or CAR T (CD45.1) cell infusion after inoculated with $CD19^+$B16. Image created with biogdp.com. (**B** and **C**) Tumor photographs (B) and tumor volumes (C) on 7 days after ACT (n = 5). (**D** and **E**) Representative flow plots (D) and histogram (E) of tumor-infiltrating host p15E-$tetramer^+CD8^+$ T cells (n = 5). Statistical significance was determined by two-tailed paired Student's t-test (C and E). *$P < 0.05$; **$P < 0.01$.

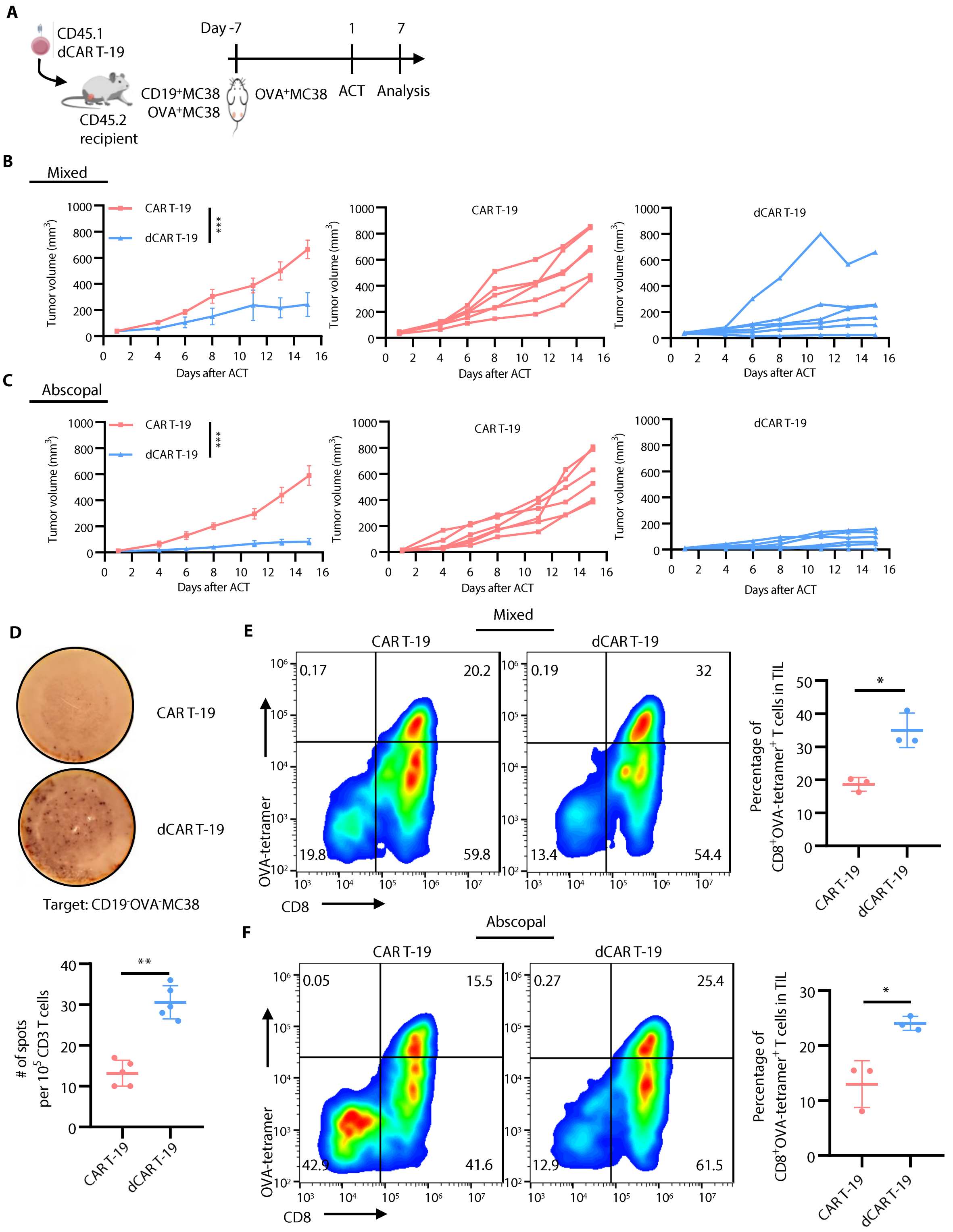

A
CD45.1 dCAR T-19
CD45.2 recipient
CD19+MC38 OVA+MC38
OVA+MC38
Day -7
1
7
ACT
Analysis
B
Mixed
CAR T-19
dCAR T-19
***
Tumor volume (mm3)
Days after ACT
C
Abscopal
D
CAR T-19
dCAR T-19
Target: CD19-OVA-MC38
# of spots per 10^5 CD3 T cells
**
E
Mixed
OVA-tetramer
CD8
0.17
20.2
19.8
59.8
0.19
32
13.4
54.4
Percentage of CD8+OVA-tetramer+ T cells in TIL
*
F
Abscopal
0.05
15.5
42.9
41.6
0.27
25.4
12.9
61.5

Fig. S8. Heterogeneous MC38 tumors were effectively controlled through antigen spreading mediated by dCAR T cells in immunocompetent mice

(**A**) Schematic of in vivo experimental design. C57BL/6 (CD45.2) mice were administered dCAR T or CAR T (CD45.1) cell infusion after inoculated with $CD19^{+}$MC38 and OVA^{+}MC38 mixed tumors in the left flank and OVA^{+}MC38 abscopal tumors in the right flank. Image created with biogdp.com. (**B** and **C**) Mixed (B) and abscopal (C) tumor growth in mice after ACT (n = 6). (**D**) IFN-γ ELISPOT (n = 3). (**E**) Representative flow plots and histogram of tumor-infiltrating OVA $tetramer^{+}$ $CD8^{+}$ T cells in mixed tumor masses on day 7 after ACT (n = 3). (**F**) Representative flow plots and histogram of tumor-infiltrating OVA $tetramer^{+}$ $CD8^{+}$ T cells in abscopal tumor masses on day 7 after ACT (n = 3). Statistical significance was determined by two-way ANOVA (B and C) or two-tailed paired Student's t-test (D to F). *$P < 0.05$; **$P < 0.01$; ***$P < 0.001$.

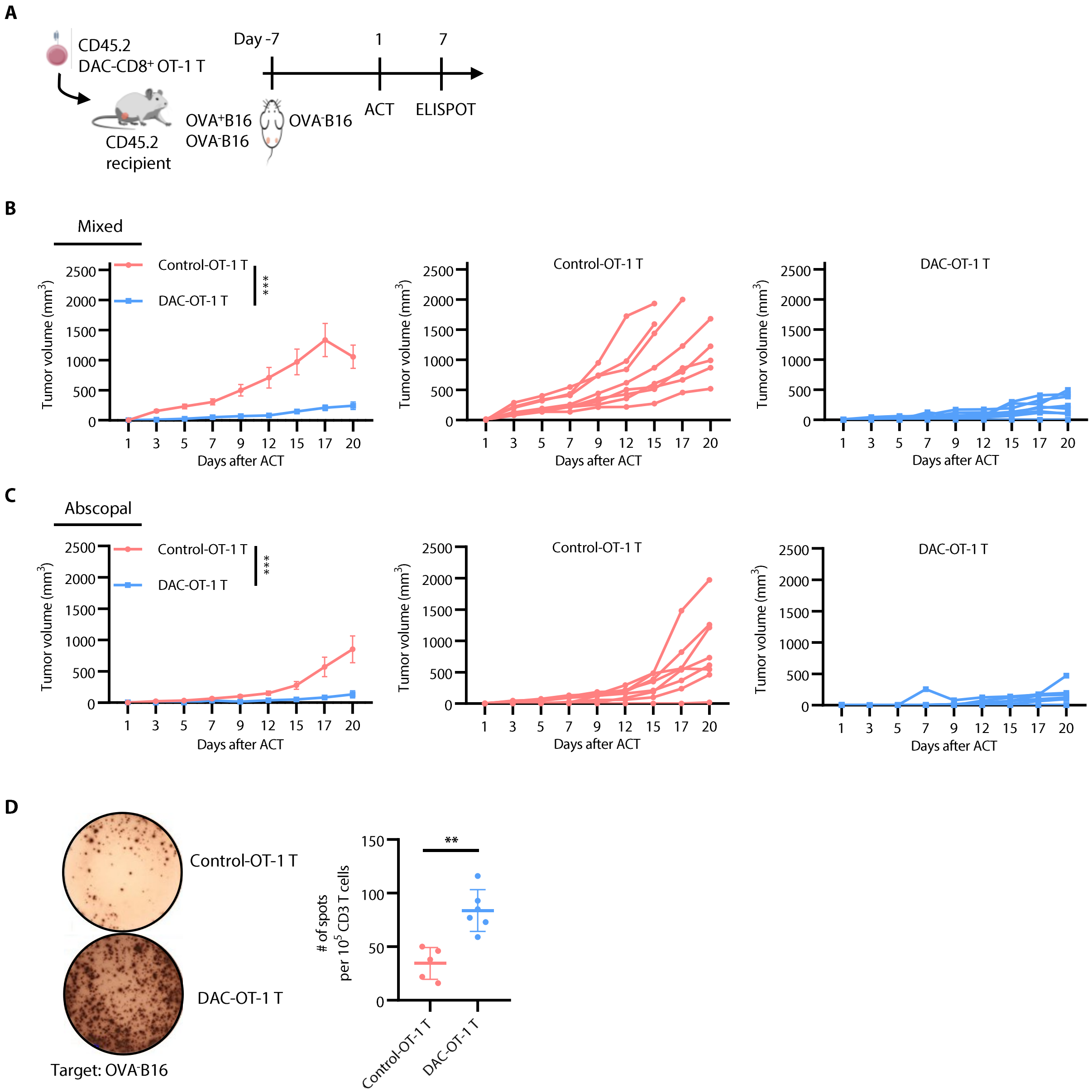
A
CD45.2
DAC-CD8+ OT-1 T
CD45.2
recipient
OVA+B16
OVA-B16
OVA-B16
Day -7
1
7
ACT
ELISPOT
B
Mixed
Control-OT-1 T
DAC-OT-1 T
***
Tumor volume (mm3)
Days after ACT
Control-OT-1 T
DAC-OT-1 T
C
Abscopal
Control-OT-1 T
DAC-OT-1 T
***
Tumor volume (mm3)
Days after ACT
Control-OT-1 T
DAC-OT-1 T
D
Control-OT-1 T
DAC-OT-1 T
Target: OVA-B16
**
# of spots per 10^5 CD3 T cells
Control-OT-1 T
DAC-OT-1 T

Fig. S9. Heterogeneous B16 tumors were effectively controlled through antigen spreading mediated by low-dose DAC priming $CD8^+$ OT-1 T cells in immunocompetent mice

(A) Schematic of in vivo experimental design. C57BL/6 (CD45.1) mice were administered DAC primed $CD8^+$ OT-1 T (DAC-OT-1 T) or conventional $CD8^+$ OT-1 T (Control-OT-1 T) (CD45.2) cell infusion after inoculated with OVA^+B16 and OVA^-B16 mixed tumors in the left flank and OVA^-B16 abscopal tumors in the right flank. Image created with biogdp.com.com. (**B** and **C**) Mixed

(B) and abscopal (C) tumor growth in mice after ACT (*n* = 8). (**D**) IFN-γ ELISPOT (control group, *n* = 5; DAC group, *n* = 6). Statistical significance was determined by two-way ANOVA (B and C) or two-tailed unpaired Student's t-test (D). $^{**}P < 0.01$; $^{***}P < 0.001$.

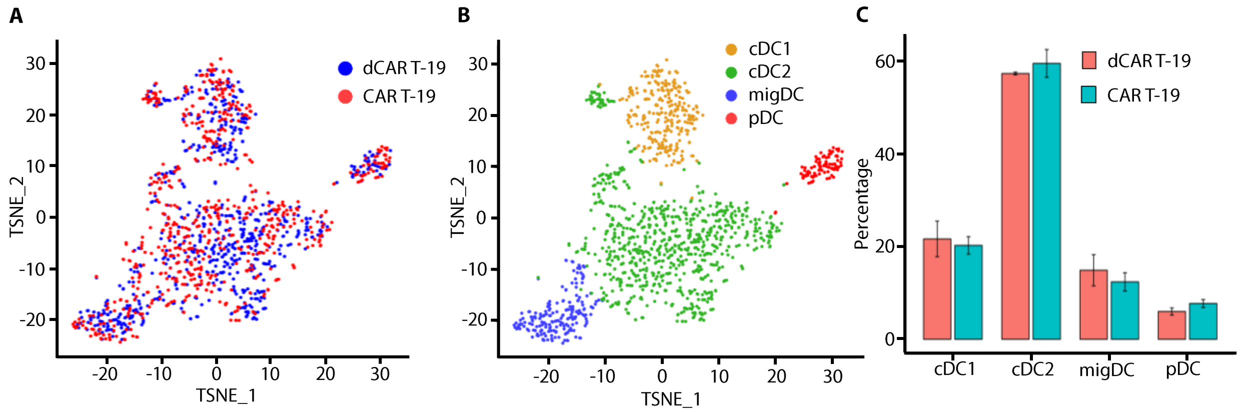

A
dCAR T-19
CAR T-19
TSNE_2
TSNE_1
B
cDC1
cDC2
migDC
pDC
TSNE_2
TSNE_1
C
dCAR T-19
CAR T-19
Percentage
cDC1
cDC2
migDC
pDC

Fig. S10. scRNA-seq analysis on tumor-infiltrating DCs

(**A**) TSNE views indicating comprehensive scRNA-seq analysis in tumor-infiltrating DCs derived from fig. S5A ($n = 2$). (**B**) Curated cell clusters for DCs based on signature gene expression ($n = 2$). (**C**) The frequency of four DCs in mice ($n = 2$).

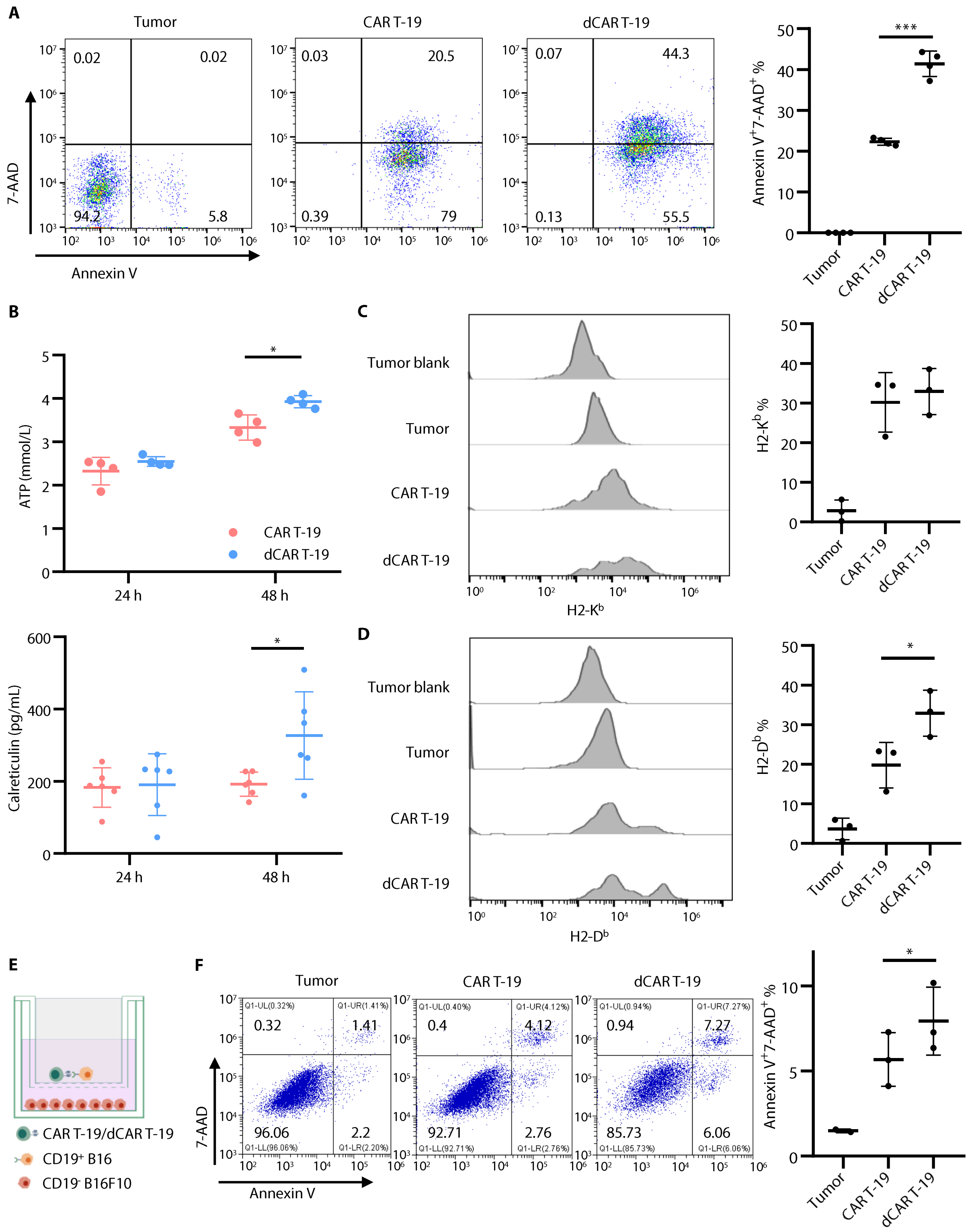

A
Tumor
CAR T-19
dCAR T-19
7-AAD
Annexin V
0.02
0.02
94.2
5.8
0.03
20.5
0.39
79
0.07
44.3
0.13
55.5
Annexin V+7-AAD+ %
***
Tumor
CAR T-19
dCAR T-19
B
ATP (mmol/L)
24 h
48 h
CAR T-19
dCAR T-19
*
Calreticulin (pg/mL)
C
Tumor blank
Tumor
CAR T-19
dCAR T-19
H2-Kb
H2-Kb %
D
H2-Db
H2-Db %
E
CAR T-19/dCAR T-19
CD19+ B16
CD19- B16F10
F
0.32
1.41
96.06
2.2
0.4
4.12
92.71
2.76
0.94
7.27
85.73
6.06

Fig. S11. Immunogenic cell death induced in tumor cells by dCAR T cells
(**A**) Flow analysis for membrane permeabilization on tumor cells after dCAR T and CAR T cells cocultured with $CD19^+$B16 cells at an E:T ratio of 1:1 for 24h (*n* = 4). (**B**) ATP (*n* = 4) and calreticulin (*n* = 6) production after dCAR T and CAR T cells cocultured with $CD19^+$B16 cells at an E:T ratio of 1:1 for 24h. (**C** and **D)** Flow analysis for H2-K^b (C) and H2-D^b (D) expression on $CD19^+$B16 tumor cells after cocultured with dCAR T and CAR T cells at an E:T ratio of 1:1 for 24h (*n* = 3). (**E**) Schematic of in vitro experimental design for (F). Image created with biogdp.com. (**F**) $CD19^-$B16 cells in lower well were analyzed membrane permeabilization by flow cytometry after cultured for 24 h (tumor group, *n* = 2; other groups, *n* = 3). Statistical significance was determined by two-tailed paired Student's t-test (A to D and F). $*P < 0.05$; $***P < 0.001$.

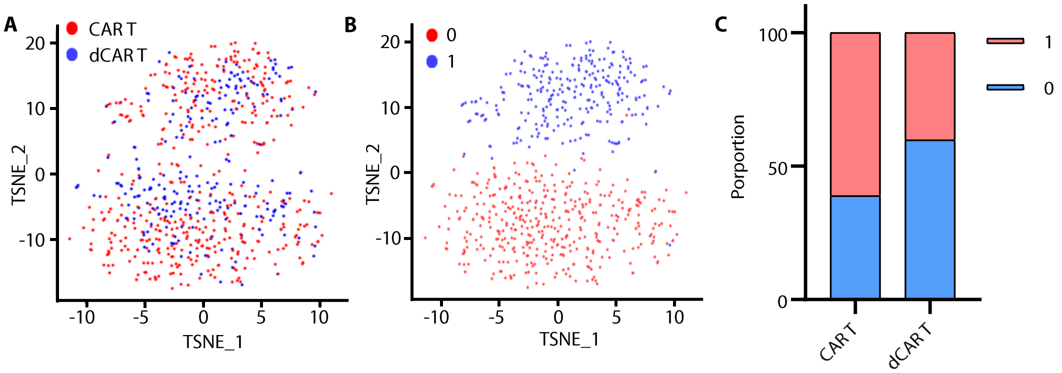
A
CAR T
dCAR T
20
10
0
-10
TSNE_2
-10
-5
0
5
10
TSNE_1
B
0
1
20
10
0
-10
TSNE_2
-10
-5
0
5
10
TSNE_1
C
100
50
0
Porportion
CAR T
dCAR T
1
0

Fig. S12. scRNA-seq analysis on tumor-infiltrating CAR$^+$ T cells

(**A**) TSNE views indicating comprehensive single-cell analysis and distinguishing tumor-infiltrating CAR$^+$ T cells derived from fig. S5A (*n* = 2). (**B**) TSNE views indicating curated cell clusters of CAR$^+$ T cells (*n* = 2). (**C**) Stacked charts showing the proportions of each tumor-infiltrating CAR$^+$ T cell cluster (*n* = 2).

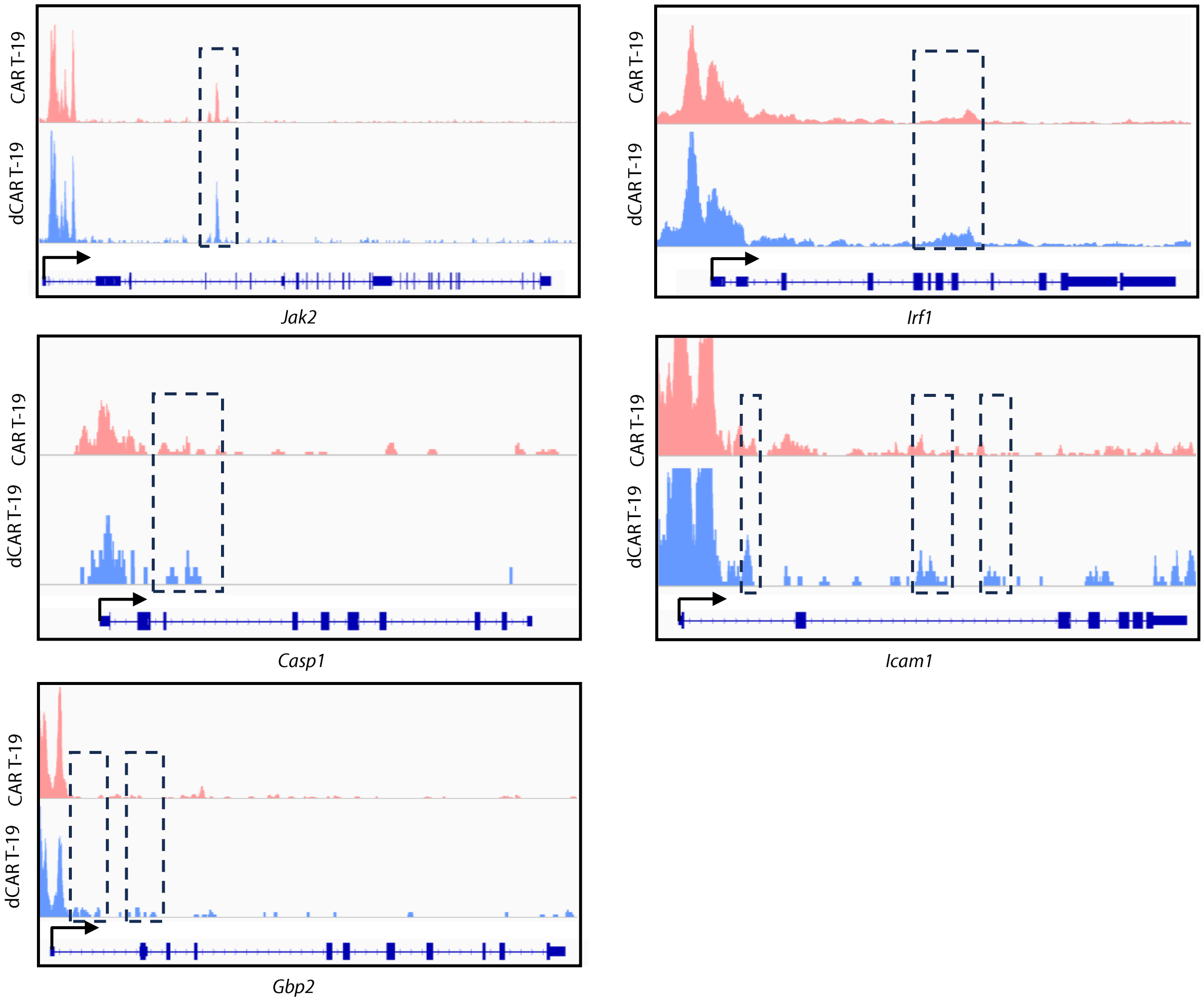
CART-19
dCART-19
Jak2
CART-19
dCART-19
Irf1
CART-19
dCART-19
Casp1
CART-19
dCART-19
Icam1
CART-19
dCART-19
Gbp2

Fig. S13. ATAC-seq analysis on in vitro cultured CAR T and dCAR T cells
Representative ATAC-seq tracks from in vitro cultured CAR T and dCAR T cells without antigen-activation at *Jak2*, *Irf1*, *Casp1*, *Icam1* and *Gbp2* regions ($n = 2$).

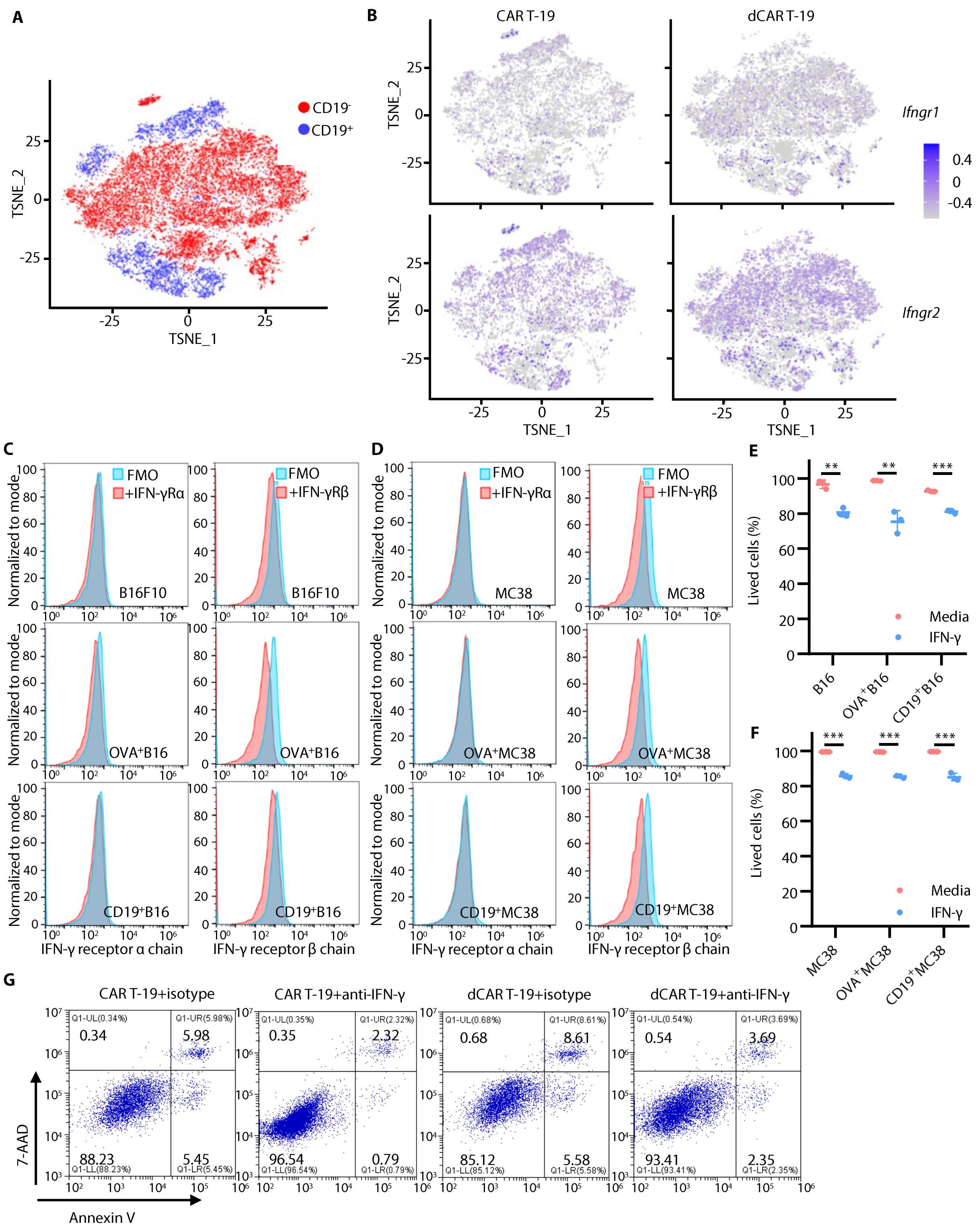
A
B
CAR T-19
dCAR T-19
Ifngr1
Ifngr2
TSNE_1
TSNE_2
CD19-
CD19+
C
D
E
F
G
FMO
+IFN-γRα
+IFN-γRβ
B16F10
OVA+B16
CD19+B16
MC38
OVA+MC38
CD19+MC38
Normalized to mode
IFN-γ receptor α chain
IFN-γ receptor β chain
Lived cells (%)
Media
IFN-γ
B16
MC38
CAR T-19+isotype
CAR T-19+anti-IFN-γ
dCAR T-19+isotype
dCAR T-19+anti-IFN-γ
0.34
5.98
88.23
5.45
0.35
2.32
96.54
0.79
0.68
8.61
85.12
5.58
0.54
3.69
93.41
2.35
7-AAD
Annexin V

Fig. S14. The expression of IFN-γ receptors on tumor cells and IFN-γ induced cell death
(**A**) TSNE views indicating comprehensive single-cell analysis and distinguishing $CD19^+$ and $CD19^-$ tumor cells derived from fig. S5A ($n = 2$). (**B**) TSNE views indicating *Ifngr1* and *Ifngr2* expression in both $CD19^+$ and $CD19^-$ tumor cells ($n = 2$). (**C** and **D**) The expression of IFN-γ receptor α chain and IFN-γ receptor β chain on B16 (C) and MC38 (D) determined by flow cytometry. (**E** and **F**) Percentage of lived cells for B16 (E) and MC38 (F) cultured in the absence or presence of IFN-γ for 48 hours ($n = 3$). (**G**) Flow analysis for membrane permeabilization of antigen-negative tumor cells in the absence or presence of anti-IFN-γ antibody in the setting attributed from in vitro experiment (Fig. 5J and K). Statistical significance was determined by two-tailed paired Student's t-test (E, F). $^{**}P < 0.01$, $^{***}P < 0.001$.

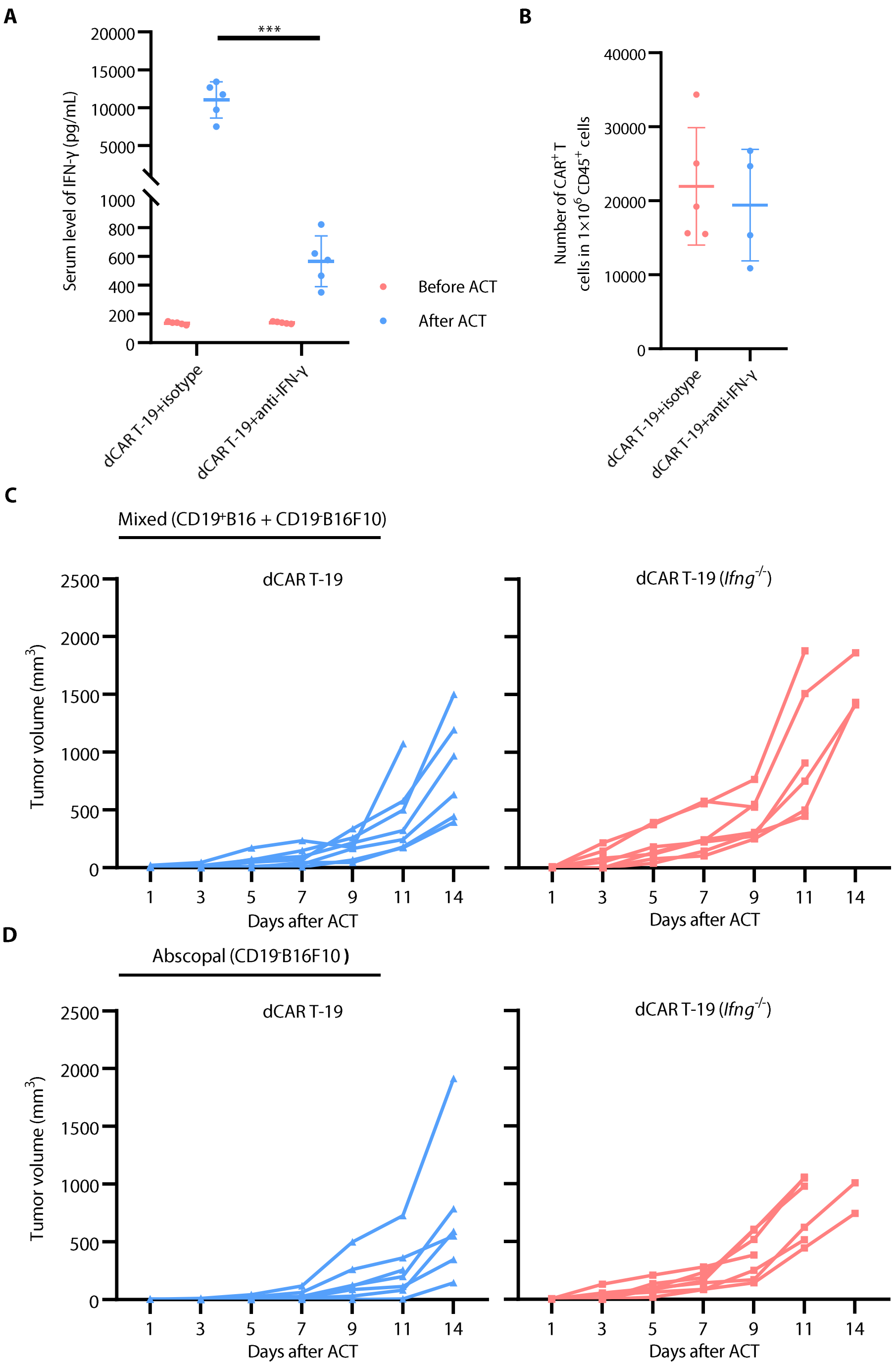
A
***
Serum level of IFN-γ (pg/mL)
20000
15000
10000
5000
1000
800
600
400
200
0
Before ACT
After ACT
dCAR T-19+isotype
dCAR T-19+anti-IFN-γ
B
Number of CAR+ T cells in 1×10^6 CD45+ cells
40000
30000
20000
10000
0
dCAR T-19+isotype
dCAR T-19+anti-IFN-γ
C
Mixed (CD19+B16 + CD19-B16F10)
dCAR T-19
dCAR T-19 (Ifng-/-)
Tumor volume (mm3)
2500
2000
1500
1000
500
0
1
3
5
7
9
11
14
Days after ACT
D
Abscopal (CD19-B16F10)
dCAR T-19
dCAR T-19 (Ifng-/-)
Tumor volume (mm3)
Days after ACT

Fig. S15. The change of serum IFN-γ level and CAR^+ T cell number following anti-IFN-γ blockade and the change of tumor growth after wide type and *$Ifng^{-/-}$* dCAR T cell therapy
(**A**) After anti-IFN-γ blockade, the serum expression levels of IFN-γ before and 8 days after dCAR T cell therapy attributed from in vivo experiment (Fig. 6A) is shown (*n* = 5). (**B**) Enumeration of tumor-infiltrating CAR^+ T cells in $CD45^+$ cells within $CD19^+$B16 and OVA^+B16 mixed tumors after anti-IFN-γ blockade (isotype group, *n* = 5; anti-INF-γ group, *n* = 4). (**C** and **D**) Mixed $CD19^+$B16 and $CD19^-$B16F10 (C) and abscopal $CD19^-$B16F10 (D) tumor growth in mice after wide type dCAR T and *$Ifng^{-/-}$* dCAR T cell therapy attributed from in vivo experiment (Fig. 6G) is shown (*n* = 7). Statistical significance was determined by two-tailed paired Student's t-test (A). *$P < 0.05$.

Table S1. Gene sequences for human CD19 and GFP.

Gene	Sequences (5’ to 3’)
human CD19	ATGCCACCTCCTCGCCTCCTCTTCTTCCTCCTCTTCCTCACCCCCATGGAAGTCAGGC<br>CCGAGGAACCTCTAGTGGTGAAGGTGGAAGAGGGAGATAACGCTGTGCTGCAGTGC<br>CTCAAGGGGACCTCAGATGGCCCCACTCAGCAGCTGACCTGGTCTCGGGAGTCCCCG<br>CTTAAACCCTTCTTAAAACTCAGCCTGGGGCTGCCAGGCCTGGGAATCCACATGAGG<br>CCCCTGGCCATCTGGCTTTTCATCTTCAACGTCTCTCAACAGATGGGGGGCTTCTACC<br>TGTGCCAGCCGGGGCCCCCCTCTGAGAAGGCCTGGCAGCCTGGCTGGACAGTCAATG<br>TGGAGGGCAGCGGGGAGCTGTTCCGGTGGAATGTTTCGGACCTAGGTGGCCTGGGCT<br>GTGGCCTGAAGAACAGGTCCTCAGAGGGCCCCAGCTCCCCTTCCGGGAAGCTCATGA<br>GCCCCAAGCTGTATGTGTGGGCCAAAGACCGCCCTGAGATCTGGGAGGGAGAGCCT<br>CCGTGTCTCCCACCGAGGGACAGCCTGAACCAGAGCCTCAGCCAGGACCTCACCATG<br>GCCCCTGGCTCCACACTCTGGCTGTCCTGTGGGGTACCCCCTGACTCTGTGTCCAGGG<br>GCCCCCTCTCCTGGACCCATGTGCACCCCAAGGGGCCTAAGTCATTGCTGAGCCTAG<br>AGCTGAAGGACGATCGCCCGGCCAGAGATATGTGGGTAATGGAGACGGGTCTGTTG<br>TTGCCCCGGGCCACAGCTCAAGACGCTGGAAAGTATTATTGTCACCGTGGCAACCTG<br>ACCATGTCATTCCACCTGGAGATCACTGCTCGGCCAGTACTATGGCACTGGCTGCTG<br>AGGACTGGTGGCTGGAAGGTCTCAGCTGTGACTTTGGCTTATCTGATCTTCTGCCTGT<br>GTTCCCTTGTGGGCATTCTTCATCTTCAAAGAGCCCTGGTCCTGAGGAGGAAAAGAA<br>AGCGAATGACTGACCCCACCAGGAGATTCTTCAAAGTGACGCCTCCCCCAGGAAGC<br>GGGCCCCAGAACCAGTACGGGAACGTGCTGTCTCTCCCCACACCCACCTCAGGCCTC<br>GGACGCGCCCAGCGTTGGGCCGCAGGCCTGGGGGGCACTGCCCCGTCTTATGGAAA<br>CCCGAGCAGCGACGTCCAGGCGGATGGAGCCTTGGGGTCCCGGAGCCCGCCGGGAG<br>TGGGCCCAGAAGAAGAGGAAGGGGAGGGCTATGAGGAACCTGACAGTGAGGAGGA<br>CTCCGAGTTCTATGAGAACGACTCCAACCTTGGGCAGGACCAGCTCTCCCAGGATGG<br>CAGCGGCTACGAGAACCCTGAGGATGAGCCCCTGGGTCCTGAGGATGAAGACTCCTT<br>CTCCAACGCTGAGTCTTATGAGAACGAGGATGAAGAGCTGACCCAGCCGGTCGCCA<br>GGACAATGGACTTCCTGAGCCCTCATGGGTCAGCCTGGGACCCCAGCCGGGAAGCA<br>ACCTCCCTGGCAGGGTCCCAGTCCTATGAGGATATGAGAGGAATCCTGTATGCAGCC<br>CCCCAGCTCCGCTCCATTCGGGGCCAGCCTGGACCCAATCATGAGGAAGATGCAGAC<br>TCTTATGAGAACATGGATAATCCCGATGGGCCAGACCCAGCCTGGGGAGGAGGGGG<br>CCGCATGGGCACCTGGAGCACCAGGTGA
GFP	ATGGTGAGCAAGGGCGAGGAGCTGTTCACCGGGGTGGTGCCCATCCTGGTCGAGCT<br>GGACGGCGACGTAAACGGCCACAAGTTCAGCGTGTCCGGCGAGGGCGAGGGCGATG<br>CCACCTACGGCAAGCTGACCCTGAAGTTCATCTGCACCACCGGCAAGCTGCCCGTGC<br>CCTGGCCCACCCTCGTGACCACCCTGACCTACGGCGTGCAGTGCTTCAGCCGCTACC<br>CCGACCACATGAAGCAGCACGACTTCTTCAAGTCCGCCATGCCCGAAGGCTACGTCC<br>AGGAGCGCACCATCTTCTTCAAGGACGACGGCAACTACAAGACCCGCGCCGAGGTG<br>AAGTTCGAGGGCGACACCCTGGTGAACCGCATCGAGCTGAAGGGCATCGACTTCAA<br>GGAGGACGGCAACATCCTGGGGCACAAGCTGGAGTACAACTACAACAGCCACAACG<br>TCTATATCATGGCCGACAAGCAGAAGAACGGCATCAAGGTGAACTTCAAGATCCGC<br>CACAACATCGAGGACGGCAGCGTGCAGCTCGCCGACCACTACCAGCAGAACACCCC<br>CATCGGCGACGGCCCCGTGCTGCTGCCCGACAACCACTACCTGAGCACCCAGTCCGC<br>CCTGAGCAAAGACCCCAACGAGAAGCGCGATCACATGGTCCTGCTGGAGTTCGTGA<br>CCGCCGCCGGGATCACTCTCGGCATGGACGAGCTGTACAAGTAA
